# Oral microbiota and periodontitis severity among Hispanic adults

**DOI:** 10.3389/fcimb.2022.965159

**Published:** 2022-11-14

**Authors:** Ana P. Ortiz, Kimil T. Acosta-Pagán, Carla Oramas-Sepúlveda, Maira A. Castañeda-Avila, Brayan Vilanova-Cuevas, Jeslie M. Ramos-Cartagena, José A. Vivaldi, Josué Pérez-Santiago, Cynthia M. Pérez, Filipa Godoy-Vitorino

**Affiliations:** ^1^ Division of Cancer Control and Population Sciences, University of Puerto Rico Comprehensive Cancer Center, San Juan, Puerto Rico; ^2^ Graduate School of Public Health, Medical Sciences Campus, University of Puerto Rico, San Juan, Puerto Rico; ^3^ Department of Microbiology and Medical Zoology, Medical Sciences Campus, University of Puerto Rico, San Juan, Puerto Rico; ^4^ University of Massachusetts Chan Medical School, Population and Quantitative Health Science, Worcester, MA, United States; ^5^ University of Puerto Rico, Medical Sciences Campus, UPRMDACC Partnership for Excellence in Cancer Research Program, San Juan, Puerto Rico; ^6^ School of Dental Medicine, Recinto Gurabo, Universidad Ana G. Mendez, Gurabo, Puerto Rico

**Keywords:** oral cavity, microbiota, 16S rRNA, periodontitis (inflammatory), hispanics

## Abstract

**Background:**

Periodontitis, one of the most common bacterial infections characterized by chronic inflammation, is also known to be a risk factor for chronic conditions, including cardiovascular disease and cancer. This inflammation is driven by an altered microbiota with an increase in pathogenic bacteria. We evaluated the association between oral microbiota and periodontitis severity in high-risk Hispanics.

**Method:**

This cross-sectional study recruited 134 sexually active participants aged 21 to 49 years old from STI Clinics in Puerto Rico. A periodontal examination, saliva collection, and an interviewer-administered questionnaire were performed. Periodontal severity was categorized as: having no disease, mild, and moderate/severe and BOP and tooth loos was noted. Saliva samples were collected for genomic DNA extraction, downstream 16S rDNA amplification sequencing, and bioinformatics analyses.

**Results:**

The structure, composition, and diversity of bacterial communities differed significantly according to periodontal severity. The richness and overall diversity also differed between participants without periodontitis and participants with some level of periodontal disease. A higher abundance of *Prevotella, Veillonella*, or *Treponema* was attributed to periodontal disease and *Aggregatibacter* to severe bleeding on probing, while *Neisseria* was found in higher abundance in healthy participants, decreasing its levels with drinking, smoking, and oral sex practices.

**Conclusions:**

Our findings indicate that dysbiosis occurs as periodontal disease progresses, and both alcohol consumption and smoking habits pose risk factors for oral dysbiosis. These results are of public health and clinical impact, as several bacteria identified could serve in the future as biomarkers for periodontitis and oral cancer risk.

## Introduction

In the United States, 64.7 million adults aged 30 years and older suffer from periodontal diseases such as gingivitis or periodontitis (Eke et al., 2012). Periodontitis is an inflammatory immune response caused by microbial dysbiosis of the dental plaque surrounding the gingiva (Genco and Borgnakke, 2013), which can lead to chronic inflammation and eventual destruction of the bone and tissue supporting the teeth. In general terms, about 20% to 50% of the human population suffers from periodontal diseases (Nazir, 2017). Periodontitis initiates with the inflammation of the gums, a disease called gingivitis (Michaud et al., 2017). In time, with the accumulation of dental plaque, microbial dysbiosis, gingival recession, destruction of the tissue, and alveolar bone loss may advance to periodontitis, chronic and irreversible inflammation of the tissue that surrounds and supports the teeth (Michaud et al., 2017). While studies have confirmed gingivitis progresses to periodontitis, this this is not always the case (Shaw et al., 2016). Rooting and scaling are periodontal treatments that may reduce the conversion of gingivitis to periodontitis (Manresa et al., 2018). However, once the tissue is damaged and the bone is lost, these changes become permanent (Michaud et al., 2017).

The oral microbiota, an essential part of the human microbiota, includes all kinds of microorganisms that reside in the oral cavity (Deo and Deshmukh, 2019). In humans, the oral cavity contains approximately 700 different bacterial species, much of them commensal and necessary to preserve the balance of the human oral physiology (Dewhirst et al., 2010). The presence of an ecological imbalance of a microbial community, called dysbiosis, in the oral cavity can cause or play a role in the development of diseases such as gingivitis and periodontitis (Mascitti et al., 2019). Poor oral hygiene and periodontal disease have been associated with oral cancer. More specifically, it has been evidenced by the association between oral dysbiosis and head and neck cancer development (Mascitti et al., 2019). Despite advances in cancer therapies and treatments, rates of head and neck and oropharyngeal cancers are increasing, particularly among Hispanics (Suárez et al., 2009). A high prevalence of high-risk sexual practices in the adult population in Puerto Rico has been reported: 82.2% of men and 77.4% of women in Puerto Rico reported having ever engaged in oral sex, 37.8% of men and 21.4% of women reported sexual initiation ≤15 years, and 47.9% of men and 13.2% of women reported a history of at least seven sexual partners in their lifetime, with a higher prevalence of these behaviors in younger adults (Ortiz et al., 2011). Additionally, among drug users aged 18-35 years living in Puerto Rico and attending the STI/Immunology Clinic of San Juan, a high prevalence of oral HPV infection (12.6%) was reported (Suárez et al., 2009). Hispanics have been characterized as having higher rates of HPV infections and oral cancer both which have been associated to periodontal disease.

We hypothesized that changes in the oral bacterial communities in participants coming to STI Clinics, would be associated to periodontal severity. Changes in the microbiota associated to periodontitis risk factors including smoking and alcohol consumption are also expected. To test this hypothesis we aimed to characterize the oral microbiota among Hispanic adults living in Puerto Rico and evaluated its association to periodontitis.

## Materials and methods

### Study population

This cross-sectional study of a convenience sample included 134 Hispanic adults aged 21 to 49 years recruited specifically from sexually transmitted infections (STI) clinics of Puerto Rico, a cohort of sexually active adults, at a higher risk of developing other STIs as they are visiting the clinics with STI related concerns such as HPV infections or HIV.

Inclusion criteria included being sexually active, having four teeth or more -as applied in the SOALS protocol (Joshipura et al., 2018)- and being cognitively capable of participating in the study. Exclusion criteria included factors known to impact the composition of the human microbiome and included individuals who were pregnant, breastfeeding, hormonal contraceptive users, postmenopausal status, had a history of antibiotics use in the preceding two months, history of post-traumatic stress or depression in past 12 months, HIV positive, antibiotics prophylaxis use, history of complications associated with their joints replacement surgery, and history of the following conditions: infective endocarditis, valvular heart disease, prosthetic cardiac valves, cardiac transplant, unrepaired cyanotic congenital heart disease, and any repaired congenital heart defect. These factors have an impact on the oral microbiota that could lead to dysbiosis; therefore, we decided to establish this extensive exclusion criterion.

### Participant recruitment

This study was approved by the Institutional Review Boards of the University of Puerto Rico Comprehensive Cancer Center Protocol number: 2018-01-01, and *via* UPR Inter-Institutional (“reciprocity”) IRB Agreement. Written informed consent was provided by all subjects. Participants were recruited from two STI clinics in San Juan, Puerto Rico. Within collaborating STI clinics, participants were invited to participate in the study by research personnel and with the use of flyers. Eligible participants were invited to complete the research visit at the facilities of the Hispanic Alliance for Clinical and Translational Research. After informed consent, study procedures were completed with the support of trained research staff.

### Data collection procedures and assessment of covariates

Study procedures included an Audio Computer-Assisted Self-Interviewing (ACASI) and an interviewer-administered questionnaire which included, among other things, sexual behavior, drinking, and smoking. A trained nurse took anthropometric measurements of study participants, saliva was collected for microbiota analyses, and lastly a dentist performed a periodontal evaluation.

In detail, the interviewer-administered questionnaire collected socio-demographic characteristics, history of medical, dental, and oral comorbidities, toxic habits (smoking and alcohol consumption), and oral hygiene measures. We included the following variables in this study: sex at birth (male, female), age (21-30, 31-40, and 41-49), lifetime smoking habits (non-smoker, smoker), alcohol consumption in the last 12 months (yes/no), lifetime marihuana usage (users, non-users), self-reported oral hygiene (poor, good, excellent), and oral sex practices (yes/no). Anthropometric measurements were used to calculate body mass index (kg/m^2^) and classify participants as underweight (BMI<18.5 kg/m^2^), normal weight (18.5-24.9 kg/m^2^], overweight (25.0-29.9 kg/m^2^), and obese (≥30 kg/m^2^).

### Periodontal assessment

A full-mouth periodontal examination was performed following CDC’s National Health and Nutrition Examination Survey (NHANES) protocol (National Center for Health Statistics, 2018). One examiner who had been previously calibrated by (NHANES) reference examiner conducted the periodontal assessments.

Periodontal disease was defined according to the Centers for Disease Control/American Academy of Periodontology (CDC/AAP). Periodontitis severity was assessed by clinical measurements of probing depth (PD) and clinical attachment loss (AL) at six sites (distobuccal, mid-buccal, mesiobuccal, disto-lingual, mid-lingual, and mesio-lingual buccal) for all teeth, excluding the third molars. All measurements were taken with a periodontal probe and rounded off upwards to the nearest millimeter. Missing teeth were noted. The NHANES reference examiner (Dr. Bruce Dye) trained and calibrated the examiner. The Centers for Disease Control/American Academy of Periodontology (CDC/AAP) working definition was used to define severe periodontitis (≥2 interproximal sites with CAL≥6 mm (not on the same tooth) and≥1 interproximal site with PD≥5 mm). Moderate periodontitis was defined as (≥2 interproximal sites with CAL≥4 mm (not on the same tooth) or ≥2 interproximal sites with PD≥5 mm (not on the same tooth), while mild periodontitis was defined as (≥2 interproximal sites with CAL≥3 mm and ≥2 interproximal sites with PD≥4 mm (not on the same tooth) or≥1 site with PD≥5 mm. Periodontal disease severity was categorized as no, mild, and moderate/severe for analytical purposes of the microbiota profiles. Periodontal status was categorized as healthy and periodontitis. Bleeding on probing (BOP) as an inflammatory parameter was also calculated. About 20 seconds after probing, BOP was marked as present if bleeding was detected at the lingual and/or buccal surfaces respectively. BOP was classified as high for each individual if 30% or more of buccal and/or lingual surfaces showed BOP (Joshipura et al., 2018) and for the purpose of severity categories we used BOP severity categories we used for a range of no BOP (0-9%), mild (10-29%), moderate (30-50%) and severe (>50%).

### Oral microbiota collection and analysis

Saliva collection was done prior to probing and periodontal examination and participants were asked to refrain from eating, drinking, or using a mouthwash for at least 2 hours before saliva collection. Unstimulated saliva (5 mL) was collected using sterile suction tubes to aid in its collection. Samples were stored on a -80C freezer until genomic DNA extraction.

The applied laboratory protocols were approved by the University of Puerto Rico Biosafety (IBC) protocol # 49218. Genomic DNA was extracted using the Qiagen PowerSoil Kit (QIAGEN LLC, Germantown Road, Maryland, USA) optimized protocol. For sample preparation, we collected 1.0mL of saliva in a 1.5mL centrifuge tube and centrifuged the tube at 13.2 rpm for 5 minutes, discarding the supernatant while the pellet was kept for the extraction. DNA was extracted from the saliva pellets using a standard protocol of the PowerSoil Kit, with an elution step in which we added 100uL of warmed (to 55 ^0^C) C6 solution (to the center of the filter membrane and centrifuged at maximum speed for 30 seconds at room temperature). Genomic DNA was quantified using the Qubit^®^ dsDNA HS (High Sensitivity) Assay (ranging from 5-100ng/ul) at room temperature (Waltham, Massachusetts, U.S.) and stored at - 20°C until genomic DNA amplification and sequencing.

### Amplification and 16S rRNA gene sequencing

The V4 region of the 16S ribosomal RNA gene was amplified from the previously extracted genomic DNA using the universal bacterial primers: 515F (5′-GTGCCAGCMGCCGCGGTAA-3′) and 806R (5′-GGACTACHVGGGTWTCTAAT-3′) following the Earth Microbiome Project protocols (Caporaso et al., 2012). Sequencing was outsourced using Illumina MiSeq MiSeq Reagent kit 2 x 250 at the Louisianna State University genomics facility.

### Quality control and filtering

Sequences of the 16S V4 region were deposited and processed in QIITA (Gonzalez et al., 2018), project ID 13193, using stringent quality control (Phred>30). Reads were trimmed to 250bp and clustered into operational taxonomic units (OTUs) with a 97% identity threshold. Taxonomy was assigned using the SILVA reference database (Quast et al., 2013). We only included samples with more than 6,875 reads for downstream analyses. Filtering was done in QIIME2 (Bolyen et al., 2019), where singletons (OTUs of less than three reads), reads identified as chloroplasts, mitochondria, and archaea were removed.

### Bacterial community beta and alpha diversity analyses

Bacterial community composition and beta diversity distances were analyzed by computing the pairwise Bray–Curtis distances between samples and plotted using the R package phyloseq (McMurdie and Holmes, 2013). Global differences in bacterial community composition were visualized in Rstudio (RStudio Team, 2020) with Non-metric Multidimensional Scaling (NMDS) using the command “physeq.ord <- ordinate (physeq, “NMDS”, “bray”)”. Statistical measures for similarities and dissimilarities of communities were obtained through qiime2 commands, tests, *qiime diversity beta* and *qiime diversity beta-group-significance*. The methods used were Permanova, a permutation-based extension of multivariate analyses to a matrix of pairwise distances (Kelly et al., 2015), Permdisp, a method that measures variance/dispersion between groups (Anderson et al., 2006), and Anosim, non-parametric analysis of similarities to identify if there are significant differences between 2 or more groups (W. and Wang, 2012).

Alpha diversity measures included richness (Chao1 Index) and evenness (Shannon information index). Boxplots showing alpha diversity were built using R’s phyloseq package (RStudio Team, 2020) and ggplot2, following the “plot_richness (physeq, x = “X”, measures = c(“Chao1”, “Shannon”))” command. P-values of both richness and evenness indexes were obtained through Qiime2, using *qiime diversity alpha and qiime diversity alpha-group-significance*, scripts in python which implement Kruskal-Wallis statistical tests.

### Bacterial taxonomic profiles

Qiime2 and R packages phyloseq and ggplot2 (Wickham, 2016), were used to generate taxonomic profiles according to metadata categories. The relative abundance of the OTUs was calculated using the number of sequences in each sample divided by the sum of sequences across samples. Representation of OTUs for taxonomic plots was chosen after FDR < 0.05 correction. Each barplot was represented with taxa that had a relative abundance of 5% or higher. Taxonomic bar plots were agglomerated at different taxonomic levels, transformed to relative abundance, filtered lower abundance, and reorganized by abundance and several metadata categories using “geom_bar” to create the representative bars according to metadata variables.

### Ubiquity dot plots of smoking categories

Using a species table that only included taxa that changed significantly for smoking habits (p<0.05), we calculated the percentage of samples to which each taxon is present (proportion or ubiquity) followed by the relative abundance -calculated using the number of sequences in each sample and dividing them by the sum of sequences across all samples. The OTU table and metadata files were modified into a ubiquity matrix using phyloseq. R package *reshape2* (Wickham, 2007) was used to “melt” both files. Ubiquity dot plots showcasing the relative distribution abundance (x-axis) and distribution across the samples (ubiquity) were prepared with ggplot2 (Wickham, 2016), using “facet” to divide the plot into different panels according to metadata variables (Anderson et al., 2006).

### Linear discriminant analysis with LEfSe

Linear discriminant analysis Effect Size (LEfSe) was used to identify taxa that could serve as possible biomarkers using the non-parametric factorial Kruskal-Wallis (KW) sum-rank test, Wilcoxon rank-sum test, and Linear discriminant analysis *(*LDA). OTU table, metadata and taxonomy files were uploaded on text format to the Microbiome analyst platform (Chong et al., 2020). Default data filter and normalization was used, and Horizontal bar plots depicting LEfSe were obtained using several metadata categories. Putative biomarkers were selected considering p-value < 0.05 and LDA effect size score of 1. The abundance of OTUs was used to identify biomarkers significantly associated with the metadata categories (periodontal severity, smoking habits, alcohol consumption and oral sex practices). This means that the plotted taxa were selected as being significantly increased in abundance compared to the other group.

## Results

### Clinical characteristics

Of the 134 participants included in this analysis, 15% had moderate/severe periodontitis, while 14% had mild and 71% had no periodontal disease (**Table 1**). Compared to females, a higher percentage of males had mild (74%) and moderate/severe (70%) periodontitis. Most participants with moderate/severe periodontitis had 31 to 49 years (90%) and reported poor (40%) or good (50%) oral hygiene. A higher percentage of smokers had moderate/severe periodontitis (40%) in comparison with 26% of smokers who had mild periodontitis. A similar distribution was found for alcohol consumption, where 75% of participants with severe and 84% with mild periodontitis consumed alcohol, while only a very small percentage (21%) of the participants with some level of periodontitis did not drink alcohol in the last 12 months. The vast majority (93%) of participants reported oral sex practices (**Table 1**).

**Table 1:** Clinical and behavioral characteristics by periodontal severity categories.

Characteristics	Periodontitis																	
	No						Mild						Moderate/Severe					
	N = 95(71%)						N = 19 (14%)						N = 20 (15%)					
	N	%	Sum of sequences		Sum of OTUs		N	%	Sum of sequences		Sum of OTUs		N	%	Sum of sequences		Sum of OTUs	
			Raw	Filtered	Raw	Filtered			Raw	Filtered	Raw	Filtered			Raw	Filtered	Raw	Filtered
Sex																		
Male	53	55. 8	608523	599451	17843	12895	14	73-7	175307	170220	7278	4659	14	70.0	173680	169881	6431	4429
Female	42	442	510868	500542	15675	10293	5	26_3	76212	70847	5259	2601	6	30.0	91886	90217	2917	2013
Age																		
21-30	49	51-6	578629	566739	18481	12258	12	632	161496	153788	8833	4967	2	10.0	20484	19597	1235	776
31-40	35	36.8	40627 1	400282	11780	8535	7	36-8	90023	87279	3704	2293	10	50.0	128478	125825	4906	3487
41-49	11	11-6	134491	132972	3257	2395	0	0.0	0	0	0	0	8	40.0	116604	114676	3207	2179
Smoking																		
Non-smoker	75	78_ 9	886197	870498	26509	18191	14	73-7	193710	184824	10083	5625	12	60.0	158381	154983	5735	3944
Smoker	20	2 1-1	233194	229495	7009	4997	5	26.3	57809	56243	2454	1635	8	40.0	107185	105115	3613	2498
Alcohol consumption																		
Yes	81	85.3	944846	927518	29318	20119	16	842	2 16886	207409	11033	6248	15	75.0	193032	188854	7102	4877
No	14	14-7	174545	172475	4200	3069	3	15.8	34633	33658	1504	1012	5	25.0	72534	71244	2246	1565
Oral Hygiene																		
Good	56	58.9	646537	633688	20381	13619	6	31.6	77400	75863	2659	1843	10	50.0	123723	121267	4266	2972
Poor	24	25.3	283197	279164	8305	6103	7	36.8	97777	93345	5039	2804	8	40.0	115512	113246	4069	2842
Excellent	15	15.8	189657	187141	4832	3466	6	31.6	76342	7 1859	4839	2613	2	10.0	26331	25585	1013	628
OraI Sex Practices																		
Yes	92	96.8	1085724	1066904	32408	22392	17	89.5	2 15553	206080	11207	6435	16	80.0	212573	208307	7346	5085
None	2	2.1	17435	17007	745	519	2	10.5	35966	34987	1330	825	3	15.0	41084	40201	1502	1033
Refuses	1	1-1	16232	16082	365	277	0	0.0	0	0	0	0	1	5.0	11909	11590	500	324
BMI																		
Overweight	33	34.7	371763	366611	10509	7655	6	31.6	68490	66094	3275	2042	4	20.0	44291	42659	2385	1544
Normal weight	32	33.7	384525	375192	13428	8620	9	47.4	122794	118512	5455	3289	8	40.0	94910	93215	3437	2500
Obese	26	27.4	317870	313614	8221	5917	2	10.5	32343	28955	2889	1220	7	35.0	109689	107819	3025	2039
Underweight	4	4.2	45233	44576	1360	996	2	10.5	27892	27506	918	709	1	5.0	16676	16405	501	359
Subtotal:	95	100	1119391	1099993	33518	23188	19	100	251519	241067	12537	7260	20	100	265566	260098	9348	6442
Total	Total of Samples		Total Sum Raw Sequences		Total Sum Filtered Sequences		Total Sum RawOTUs		Total Sum FilteredOTUs									
	134		1636476		1601158		55403		36890									

Participants had a mean of 4.0 ± 6.4 missing teeth, 5.2 ± 5.5 teeth with ≥3mm pocket depth, 2.3 ± 4.1 ≥3mm teeth with attachment level, 15.0 ± 11.5 bleed teeth on probing and 12.2 ± 7.0 teeth with plaque (**Table 2**).

**Table 2:** Participant dental measurements.

Dental measurements	Number of teeth
	mean ± sd
**Missing teeth**	4.0 ± 6.4
**Total teeth**	24.4 ± 4.7
**Probing pocket depth**	
≥3mm	5.2 ± 5.5
≥4mm	1.5 ± 3.2
≥5mm	0.5 ± 2.0
≥6mm	0.2 ± 1.1
≥7mm	0.1 ± 0.5
**Clinical attachment level**	
≥3mm	2.3 ± 4.1
≥4mm	0.9 ± 2.8
≥5mm	0.4 ± 2.1
≥6mm	0.3 ± 1.4
≥7mm	0.1 ± 0.6
**Bleeding on probing**	15.0 ± 11.5
**Plaque**	12.2 ± 7.0
**Coronal caries**	0.8 ± 1.6
**Coronal restoration**	6.4 ± 4.4
**Root caries**	0.2 ± 0.9
**Root restoration**	0.2 ± 1.1

### Microbiota profiles

From a total of 1,636,476 raw reads, we analyzed 1,601,158 after filtering for chloroplasts, mitochondria, archaea, and singletons. These reads produced 36,890 OTUs from all 134 samples Statistics of the species table and their corresponding categories to be analyzed shown in **Table 1**. The categories that exhibited the highest number of reads were healthy participants, age group 21-30 years, non-smokers, participants who consume alcohol, and those who reported oral sex (**Table 1**). The sum of OTUs per periodontitis severity category is also depicted according to each clinical variable (**Table 1**). Independently of a total number of reads per category, we performed all the analyses using the same number of reads per sample, corresponding to a rarefaction level of 6,875 reads for normalization purposes.

### Community diversity according to periodontal severity and bleeding on probing

**Figure 1:**
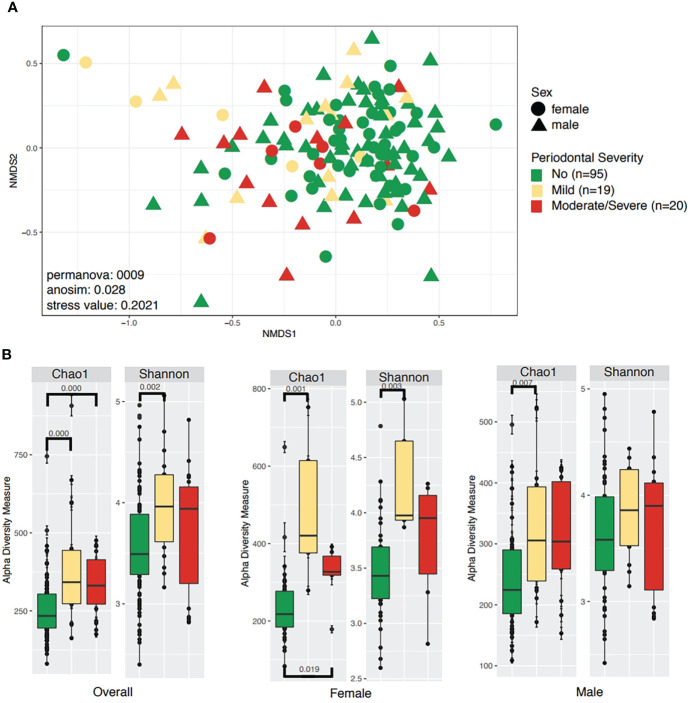
Community structure and diversity analyses according to periodontal severity and sex. **(A)** NMDS plot depicting sample distances according to periodontal severity and sex. **(B)** Boxplots for the same metadata variables above, showing species richness (Chao1) and diversity of species (Shannon). Only significant differences are shown on alpha diversity plots.

Microbiota diversity and structure, were evaluated by periodontal disease severity, comparing participants without periodontitis to those with mild and severe/moderate periodontitis (**Figure 1**). Analyses of periodontal status (healthy vs. periodontitis) are available as **Supplementary Information** (**Supplemental Figure 1**). Significant differences were found for beta diversity as shown by the separation on the beta diversity plot among those with moderate/severe periodontitis towards negative values on axis one. Permanova and Anosim tests showed a statistically significant difference in distance and composition (p<0.05) between groups (**Figure 1A**). No statistical difference in beta diversity was found when evaluating periodontal status (healthy vs. periodontitis) (**Supplementary Figure 1**; **Supplementary Table 1**).

Alpha diversity was significantly higher in participants with mild periodontitis (Shannon: 0.002). When discriminating for sex, we observed that women and men with mild periodontitis had different microbial diversity (**Figure 1B**). Additionally, a difference in richness (Chao 1) was found between those without periodontitis compared to participants with mild periodontitis (Chao1 p-value: 0.001), as well as between participants without periodontitis compared to those with moderate/severe periodontitis (Chao1 p-value: 0.001, **Figure 1B**). Regarding periodontitis disease status, we found significant differences in alpha richness (Chao1 p-value: 0.001) and diversity (Shannon p-value: 0.004) in patients with the disease compared to healthy participants (**Supplementary Figure 1B**).

**Figure 2:**
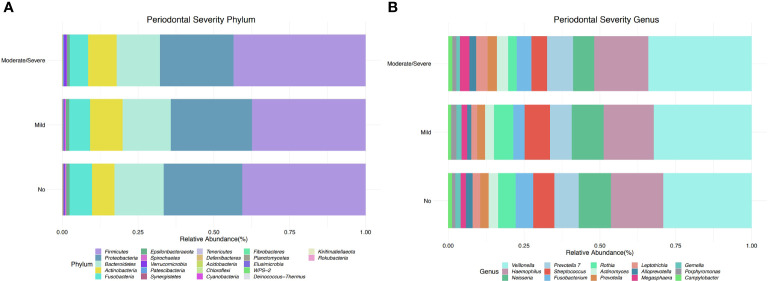
Taxonomic profiles of bacterial diversity by periodontal severity at phylum and genus level. **(A)** Taxonomic bar plot at phylum level, showing relative abundance of the most dominant taxa (FDR < 0.05). **(B)** Taxonomic bar plot at genus level, depicts relative abundance of the most dominant taxa (p-value < 0.05).

The taxonomic profiles, including taxa selected with a p-value <0.05, depict 22 different phyla, of which five were the most dominant, including Firmicutes, Proteobacteria, Bacteroidetes, Actinobacteria, and Fusobacteria (**Figure 2A**). Participants with mild periodontitis had a higher relative abundance of Proteobacteria, Bacteroidetes, and Actinobacteria, when compared with those with severe disease (**Figure 2A**). When evaluating periodontitis status, no differences were found in taxonomic composition (**Supplementary Figure 1C**). In addition, a total of 32 significant genera were detected (p-value <0.05), among which *Haemophilus, Porphyromonas, Prevotella, Ruminococcaceae*, and *Rothia* were the most abundant across all samples. *Haemophilus* abundance was higher in participants without periodontal disease, while participants with mild periodontal disease had a higher abundance of *Akkermansia, Leptotrichia*, and *Parabacteroides* (**Figure 2B**). Participants with moderate/severe periodontitis decreased *Rothia*, *Haemophilus*, and *Parobacteroides;* however, *Prevotella 7, Alloprevotella, Fusobacterium*, and *Porphyromonas* were increased in this group (**Figure 2B**). Similar findings are shown when evaluating periodontal status (**Supplementary Figure 1D**).

**Figure 3:**
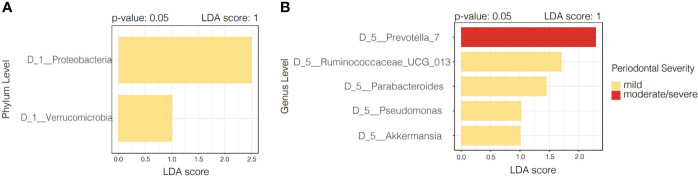
Linear discriminant analysis Effect Size (LEfSe) according to periodontal severity. **(A)** Illustrates distinctive taxa at phylum level (p < 0.05), while panel **(B)** shows distinctive taxa at genus level according to periodontal severity.

The linear discriminant analysis Effect Size (LEfSe), by periodontitis severity, demonstrates that both Proteobacteria and Verrucomicrobia were significantly more dominant in participants with mild periodontitis. Specific genus level designations are numbered such as the case of *Prevotella_7*. This is a generic designation for genus-level groups/clusters of sequences that previously fell into *Prevotella* (uncultured) to increase the resolution of taxa within family Prevotellaceae. *Prevotella_7 was* found to be distinctive in participants with moderate/severe periodontitis, while *Ruminococcaceae, Parabacteroides, Pseudomonas*, and *Akkermansia* were distinctive in those with mild periodontitis (**Figure 3**).

**Figure 4:**
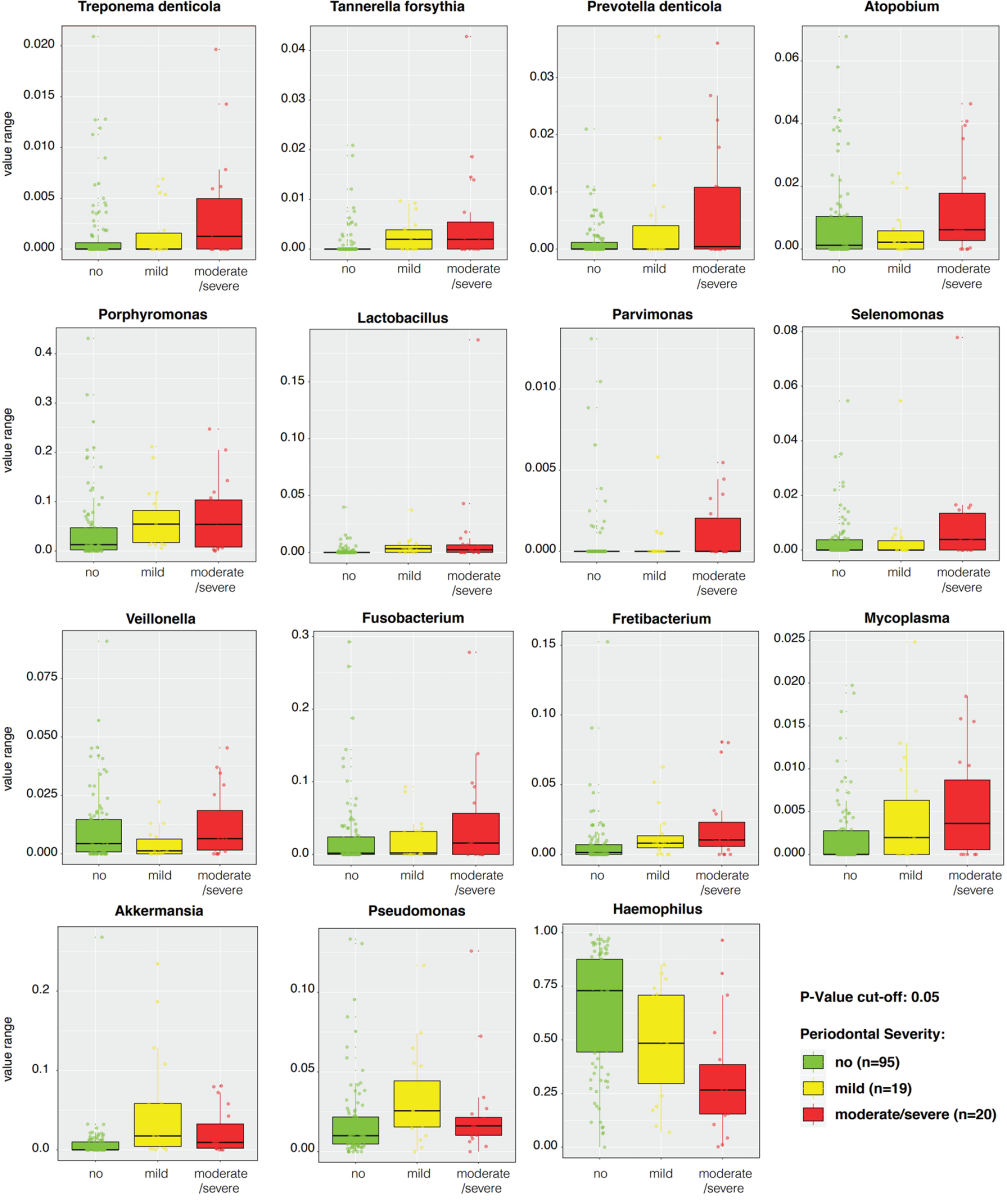
Linear discriminant analysis Effect Size (LEfSe) boxplots of significantly distinctive genera and species according to periodontal severity with a p-value cut-off < 0.05.

**Figure 5:**
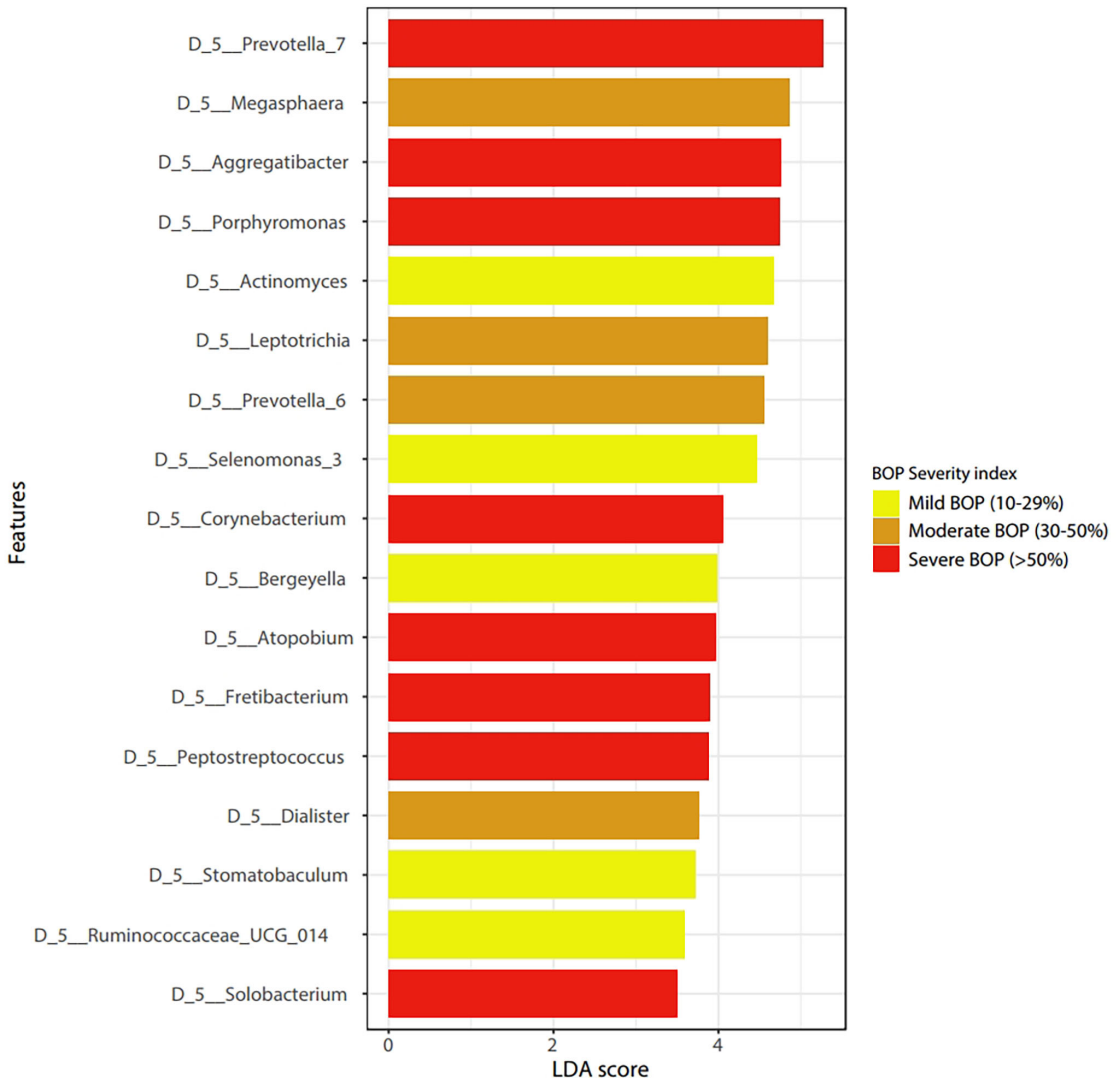
Linear discriminant analysis Effect Size (LEfSe) according to Bleeding on Probing severity at the genus level (p < 0.05).

**Figure 6:**
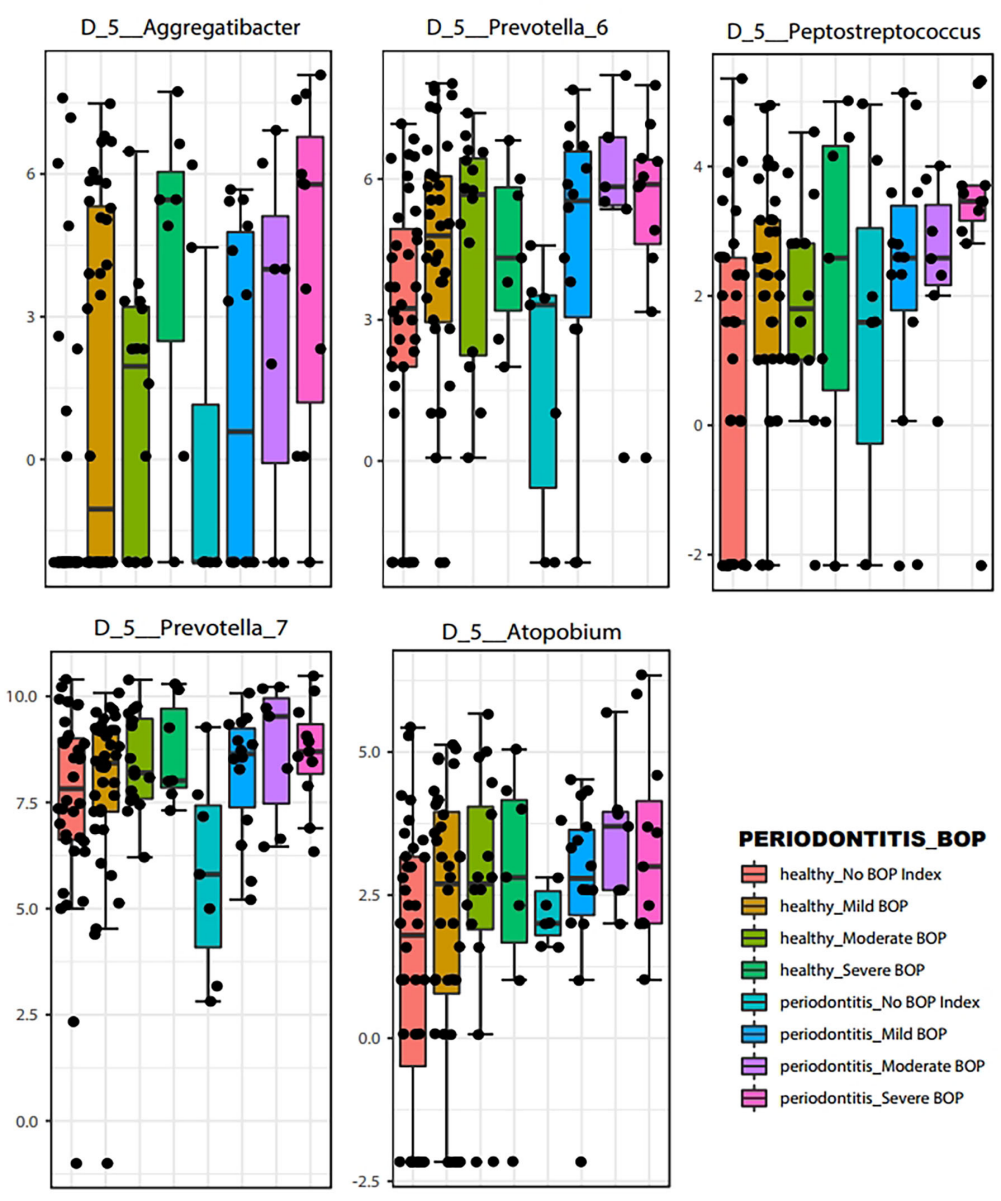
Boxplots of representative bacteria identified by LEfSe to display the microbial differences according to Bleeding on Probing severity at the genus level (p < 0.05).

LEfSe features across periodontal severity (p-value < 0.05), at genus and species levels, included *Treponema denticola, Prevotella denticola, Atopobium, Parvimonas, Selenomonas, Lactobacillus*, and *Fretibacterium* were more abundant in participants with moderate/severe periodontitis. We also confirmed that *Mycoplasma* and *Fusobacterium* increased as periodontal disease progressed, meanwhile *Haemophilus* decreased. Both *Akkermansia* and *Pseudomonas* were more abundant in participants with mild periodontitis (**Figure 4**). Bleeding on probing (BOP) addresses the percentage sites which bleed on probing, informing on inflammation and the subject’s disease progression risk. Participants with mild BOP had a mostly *Actinomyces, Selenomonas, Bergeyella* or *Stomatobaculum.* Participants with moderate bleeding (30-50%) had mostly Megasphaera, Leptotrichia and *Prevotella*_6 or *Dialister.* Those with severe bleeding (>50%) with highest levels of inflammation had a dominance of *Prevotella*_7, *Aggregatibacter, Porphyromonas, Atopobium, Fretibacterium and Peptostreptococcus* (**Figure 5**). When combining periodontal status with BOP, we found that participants with periodontitis and severe BOP had higher levels of Aggregatibacter, Prevotella_6, *Peptostreptococcus* or *Atopobium* (**Figure 6**).

### Microbiota associated to smoking, alcohol and marihuana usage habits

**Figure 7:**
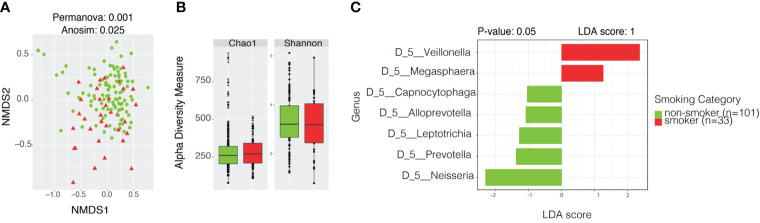
Community profiles including beta, alpha diversity, taxonomic profiles of bacterial diversity analysis and linear discriminant analysis Effect Size (LEfSe) according to smoking status. **(A)** Beta diversity analyses by Non-metric Multidimensional Scaling (NMDS) contrasting community structure according to smoking status. **(B)** Alpha diversity boxplots depicting species richness (Chao1) and overall diversity of species (Shannon) between smokers and non-smokers. **(C)** LEfSe illustrating distinctive taxa at Genus Level (p < 0.05) among smokers and non-smokers.

Microbial community analyses were also performed according to smoking status. The beta diversity analysis showed significant differences in distance among samples of smokers and non-smokers (Permanova p-value: 0.001, Anosim p-value: 0.025) but not in dispersion (Permdisp p-value: 1, **Figure 7A**). We observed a slight separation on the beta diversity plot among smokers, towards negative values at the bottom of the plot.

**Figure 8:**
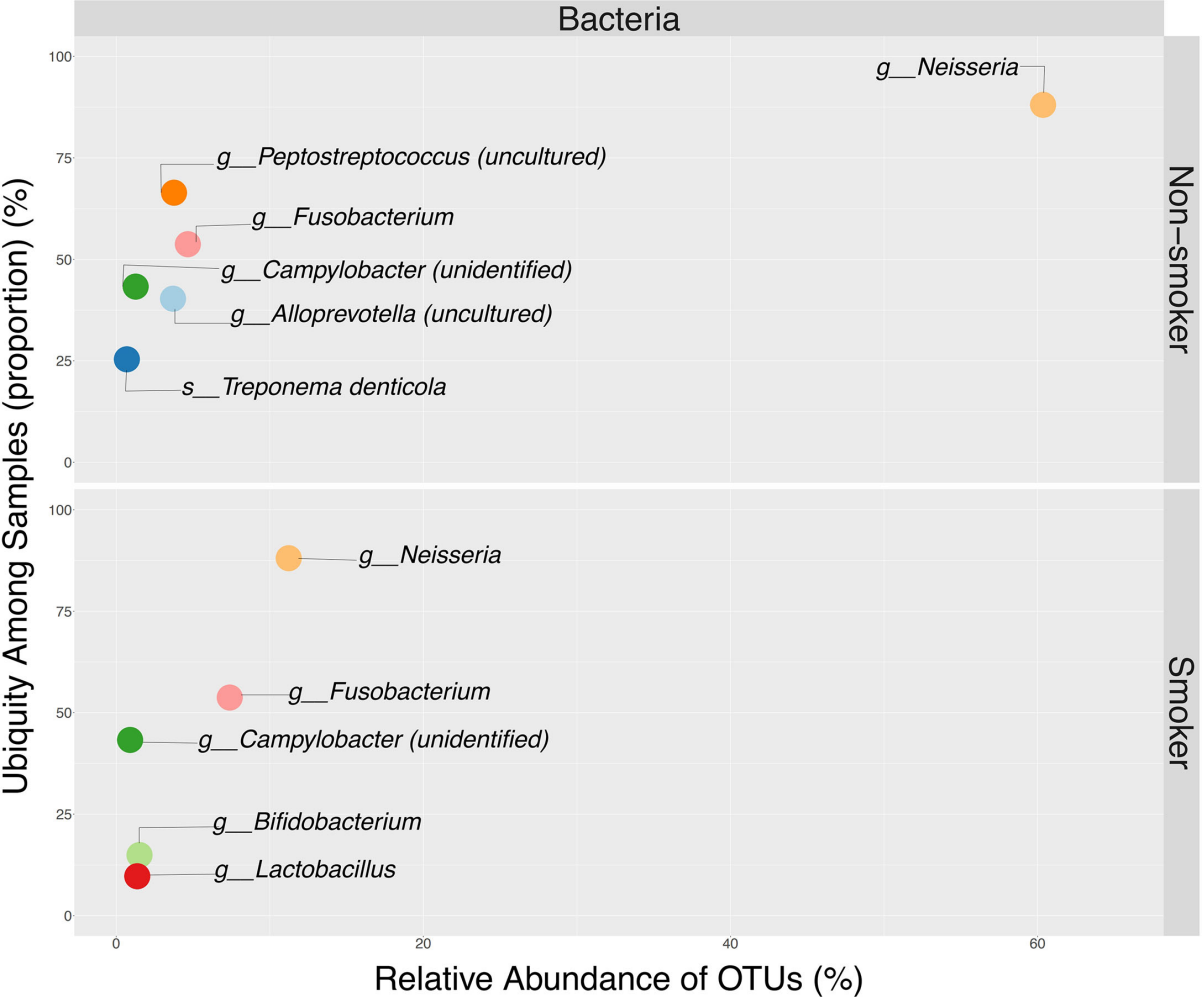
Ubiquity dot plot illustrating bacterial OTUs dominance in the oral cavity, according to their relative abundance (x-axis) and ubiquity (y-axis) by smoking status. Ubiquity was calculated by converting the OTU table into a simple column, to depict relative abundance and ubiquity using *reshape2* package. Illustration of the dot plot was obtained using *ggplot2* package available in.

Alpha Diversity analyses among smokers vs. non-smokers showed no significant differences in richness or evenness (**Figure 7B** and **Supplementary Table 2**). LEfSe analysis with a p-value<0.05 and LDA score of 1 revealed *Veillonella* and *Megasphaera* as possible biomarkers for smokers, while *Capnocytophaga, Alloprevotella, Leptotrichia, Prevotella, and Neisseria* were dominant in non-smokers (**Figure 7C** and **Supplementary Table 3**). Ubiquity plots depicting the distribution of OTUs according to relative abundance and prevalence (ubiquity) show how bacterial communities change between smokers and non-smokers. Neisseria in non-smokers had a relative abundance of ~60% while smokers decreased to ~13%. Although in low abundance, *Bifidobacterium* and *Lactobacillus* appeared in the oral cavity of smokers (**Figure 8**). The joint analyses of periodontal severity and smoking showed significant differences in bacterial composition (Permanova p-value: 0.001) (**Supplementary Figure 5A**). We also found statistically significant differences in richness (Chao1 p-value: 0.001) between non-smokers without periodontal disease and between non-smokers with mild periodontitis (**Supplementary Figure 5B**). Differences in mean diversity of species (Shannon p-value: 0.014) were only observed between non-smokers with mild or no periodontal disease (**Supplementary Figure 5B**). Statistical tests and corresponding p-values of both Chao1 and Shannon Indexes are presented in **Supplementary Table 2**. The 5 most dominant genera were *Veillonella, Haemophilus, Neisseria, Prevotella, and Streptococcus*. Smokers with periodontitis had a decrease in the abundance *of Neisseria and Streptococcus.* Both moderate/severe periodontitis categories (smokers and non-smokers) had a decrease in the abundance of *Rothia* (**Supplementary Figure 5C**).

The evaluation of microbiome changes according to marihuana usage revealed that there were significant differences in community structure (Anosim p-value: 0.033) and dispersion between samples (Permdisp p-value: 0.008) (**Supplementary Figure 2A**). No significant differences in alpha diversity were observed, although the trend supports higher diversity in marihuana-users (**Supplementary Figure 2B**). All statistical test values are shown in **Supplementary Table 2**. There were little to no differences in the taxonomic composition according to marihuana usage.

Alcohol consumption was also shown to be a modifier of the oral microbiota (**Supplementary Figure 3**). Although we did not find any significant differences in community structure (beta diversity plot), alpha (Shannon) diversity was significantly higher in the oral cavity of alcohol consumers (p-value = 0.0436) (**Supplemental Figure 3A** and **Supplementary Table 1**). Taxonomic profiles showed a decrease in the abundance of *Neisseria* and *Streptococcus* in participants who consumed alcohol (**Supplementary Figure 3C**).

Oral sexual practices did not reveal significant differences in structure, composition, richness, or overall species diversity across all categories (**Supplementary Figures 4A, B**, and **Supplementary Table 3**). Taxonomic profiles at the genus level showed that participants who practiced oral sex had higher abundance of *Haemophilus and Prevotella_7*, while the abundance of *Neisseria* decreased (**Supplementary Figure 4C**).

## Discussion

This is the first study characterizing the oral microbiota of Hispanic individuals living in Puerto Rico and coming to STI clinics, highlighting its association to periodontal disease. Our data revealed associations of the oral microbiota with periodontal disease, with concomitant differences in both composition and diversity of bacteria. We determined that as periodontal disease progressed, dysbiosis occurred comprising a reduction of commensal bacteria such as *Neisseria* and an increase in opportunistic anaerobic bacteria such as *Prevotella, Veillonella*, and *Porphyromonas* (Kumar et al., 2005; Sudhakara et al., 2018). These communities play an important role in the development of periodontal disease, due to ecological changes as their abundance shift. Dysbiosis was also attributed to other risk factors including smoking, marihuana usage and alcohol consumption, which have an impact on the oral microbiota composition and can increase the risk of developing oral cancer. Interestingly we found *Lactobacillus and Atopobium* (common vaginal bacteria) among those increasing in periodontitis or with severe BOP. Our results confirm those found in the literature, with the dominance of *Prevotella* and *Veillonella* in the saliva of patients with periodontitis, while predominance of *Neisseria* has been attributed to healthy participants (Zaura et al., 2009; Lopes et al., 2020). *Prevotella* -a taxa dominant both in periodontitis and increasing with BOP severity, has been associated to inflammation sites (Chattopadhyay et al., 2019) and has been identified in oral cancer signatures (Rovery et al., 2005; Larsen, 2017), while *Veillonella*, has been associated with serious infections such as endocarditis, osteomyelitis, and septicemia (Rovery et al., 2005). Our data suggests *Prevotella* as a possible biomarker for periodontitis and BOP confirming other reports in Hispanics (Ardila et al., 2014). In fact, *Prevotella* stimulates the inflammatory responses such as the production of CCL20, IL-8 and IL-6, aggravating inflammatory diseases (Mager et al., 2005). Other bacteria were detected as putative biomarkers for periodontitis including *Ruminococcaceae, Pseudomonas*, and *Akkermansia*, which were deemed as distinctive in participants with mild periodontal disease. *Treponema denticola, Prevotella denticola, Atopobium, and Porphyromonas* were found in participants with moderate to severe periodontal disease. *Porphyromonas* are major gram-negative pathobionts of the oral cavity associated with periodontitis. These bacteria can cause tissue damage and were found to impact systemic diseases such as Alzheimer’s, diabetes and cardiovascular diseases (Carter et al., 2017). A recent study found a higher abundance of *Treponema* and *Fretibacterium* in the saliva and subgingival plaque in participants with periodontitis and a positive correlation between *Treponema* and *Selenomonas* with inflammatory cytokines (Lundmark et al., 2019). This data suggests that an increase in genera like *Treponema, Prevotella*, and *Parvimonas* can become possible biomarkers for periodontal disease. More importantly, in participants with periodontitis, we found an increase in the abundance of *Lactobacillus and Atopobium* both belonging to the vaginal tract. This finding could imply the effect of microbial translocation from its original niche – the vagina- to the oral cavity, as *Lactobacillus* have been linked to oral HPV infections and oral cancer (Camelo-Castillo et al., 2015b; Guerrero-Preston et al., 2016; Guerrero-Preston et al., 2017).

Some of the most critical risk factors of periodontal disease and oral cancer are smoking habits and alcohol (Camelo-Castillo et al., 2015b). We found a decrease in the abundance of *Neisseria* and the appearance of *Bifidobacterium* and *Lactobacillus* in smokers. Periopathogens such as *Porphyromonas, Prevotella Tannerella* or *Treponema* have been found in significantly higher prevalence in participants with periodontal disease, especially in smokers (Camelo-Castillo et al., 2015a; Diao et al., 2021). *Aggregatibacter* is a gram-negative facultative anaerobe and an opportunistic pathogen, which has been associated with periodontitis and other inflammatory diseases (Gholizadeh et al., 2017) and here is a likely biomarker of severe BOP and periodontitis in Hispanis.

It is well established that cigarettes alter the gut composition (Vallès et al., 2018; Stewart et al., 2018; Beghini et al., 2019) and oral microbial communities (Allais et al., 2016; Yu et al., 2017), with varying health implications. Like what we found in participants with periodontitis, smoking, and alcohol consumption patterns are associated with oral dysbiosis. Toxins released from cigarette and tobacco use can impact microbes present in the oral cavity, leading to the loss of beneficial species. Alcohol might affect the pH and the stability of the oral biofilm, permitting colonization of pathogens and aiding the development of periodontal disease (Wu et al., 2016). Other research that focused on associations between alcohol consumption and oral microbiota dysbiosis found that heavy drinking influences the bacterial composition, depleting beneficial commensal bacteria while aiding colonization of pathogenic bacteria (Fan et al., 2018). However, they noticed an increase in *Neisseria* abundance with heavy alcohol consumption, unlike our findings.

## Conclusions

We acknowledge that our study had four important limitations:1) a moderate to low sample size of only 134 sexually-active adults, 2) recruitment in a younger age range (21-49 years old) as compared to older participants, as periodontitis seems to be more dominant in males over 65 years of age, 3) as periodontitis is a local inflammatory disease, saliva samples might not be sufficiently informative. And 4) lack of radiographic evaluation. Nonetheless, the detailed periodontitis phenotype, BOP and jointly with the oral dysbiosis profiles showcased the severe complications young high-risk Hispanics might face, and their vulnerability to carcinogenic morbidities. We observed that, has hypothesized, the progress of periodontal disease is associated to changes in the saliva bacterial communities. Mainly, we identified a correlation between healthy individuals and commensal bacteria, while participants with periodontal disease and with higher levels of bleeding on probing were mainly associated to opportunistic and pathogenic bacteria. Risk factors, such as smoking and alcohol consumption, were associated with an increase of opportunistic bacterial communities.

The analyses of the salivary microbiota in the routine dental care of high-risk patients could help detect severe periodontitis and prevent other complex health problems. Our findings work as a step stone to identify Hispanic biomarkers that could be used develop early diagnostic strategies for identifying oral cancer in its early stages, which could highly increase its survival rate.

Large-scale, long-term longitudinal multi-omic studies are needed identify causality of certain microbial taxa and periodontal disease progression.

## Data availability statement

The datasets presented in this study can be found in online repositories. The names of the repository/repositories and accession number(s) can be found below: EUROPEAN NUCLEOTIDE ARCHIVE https://www.ebi.ac.uk/ena/browser/text-search?query=ERP126217.

## Ethics statement

The studies involving human participants were reviewed and approved by University of Puerto Rico Comprehensive Cancer Center Protocol number 2018-01-01. The patients/participants provided their written informed consent to participate in this study.

## Author contributions

Conception and design: AO, CP and FG-V. Development of methodology: AO, CP, JV and FG-V. Acquisition of data: JV, JR-C, CO-S, KA-P. Sample processing: KA-P, BV-C. Analysis and interpretation of data: KA-P, FG-V, BV-C, JR-C and Writing, review, and/or revision of the manuscript: FG-V, AO, CP, JV, JR-C, CO-S, KA-P, BV-C, MC-A. Study supervision: AO, CP and FG-V. All authors contributed to the article and approved the submitted version.

## Funding

This project was funded by an award from the NIH National Institute of Dental and Craniofacial Research 1R21DE027226-01A1 and R21 DE027226 02S (NIDCR Diversity Supplement). Partial funds were given by the National Institute of General Medical Sciences Institutional. Development Award (IDeA) grant number 5P20GM103475; the Hispanic Alliance for Clinical and Translational Research (Alliance) National Institute of General Medical Sciences (NIGMS) U54GM133807 and the Center for Collaborative Research in Minority Health and Health Disparities 3U54MD007600-35S2.

## Conflict of interest

The authors declare that the research was conducted in the absence of any commercial or financial relationships that could be construed as a potential conflict of interest.

## Publisher’s note

All claims expressed in this article are solely those of the authors and do not necessarily represent those of their affiliated organizations, or those of the publisher, the editors and the reviewers. Any product that may be evaluated in this article, or claim that may be made by its manufacturer, is not guaranteed or endorsed by the publisher.
