# Supplementary figures and images for: Oral microbiota and periodontitis severity among Hispanic adults
Source: Front Cell Infect Microbiol. 2022 Nov 14;12:965159. doi: 10.3389/fcimb.2022.965159 (PMC9703052; doi:10.3389/fcimb.2022.965159)

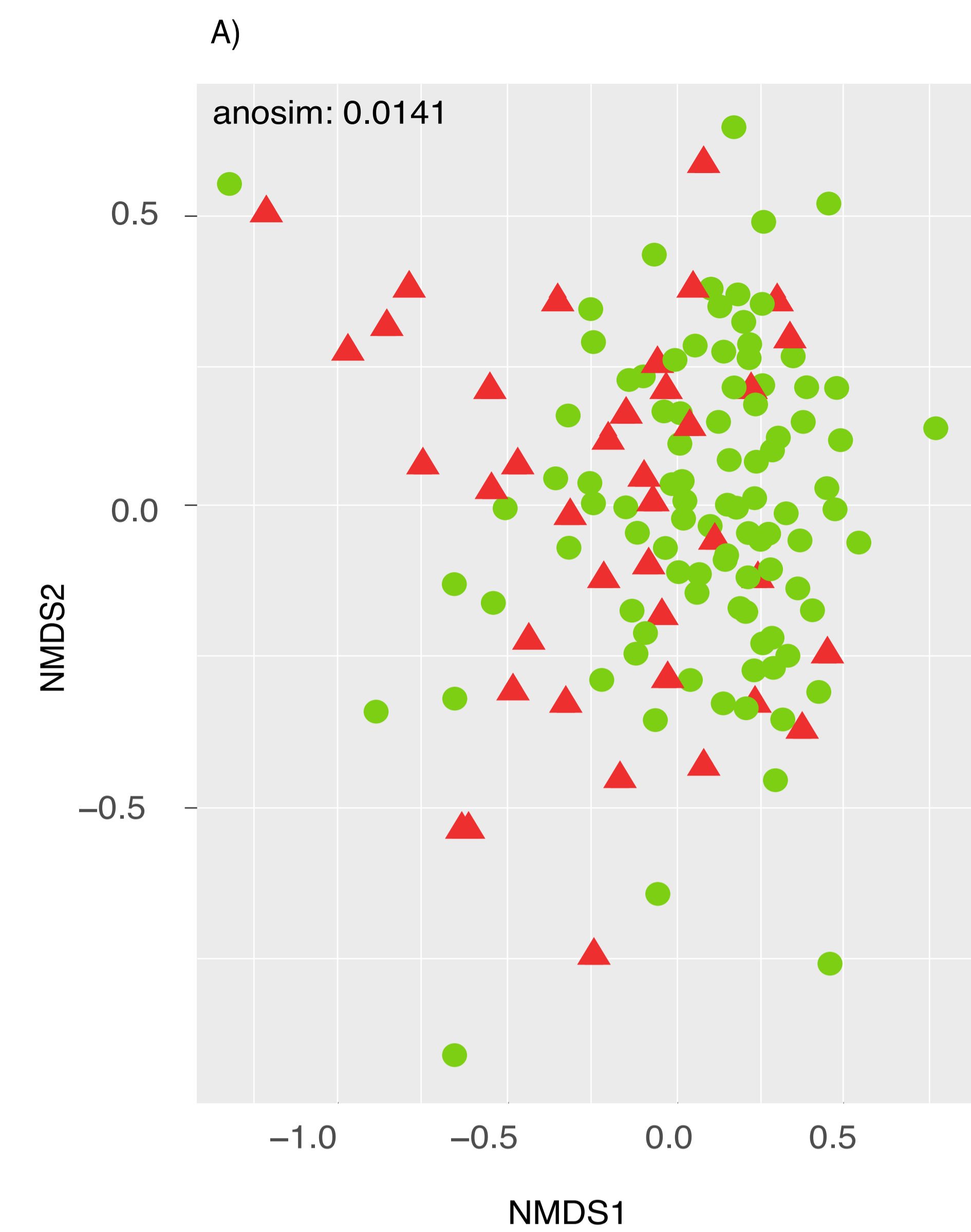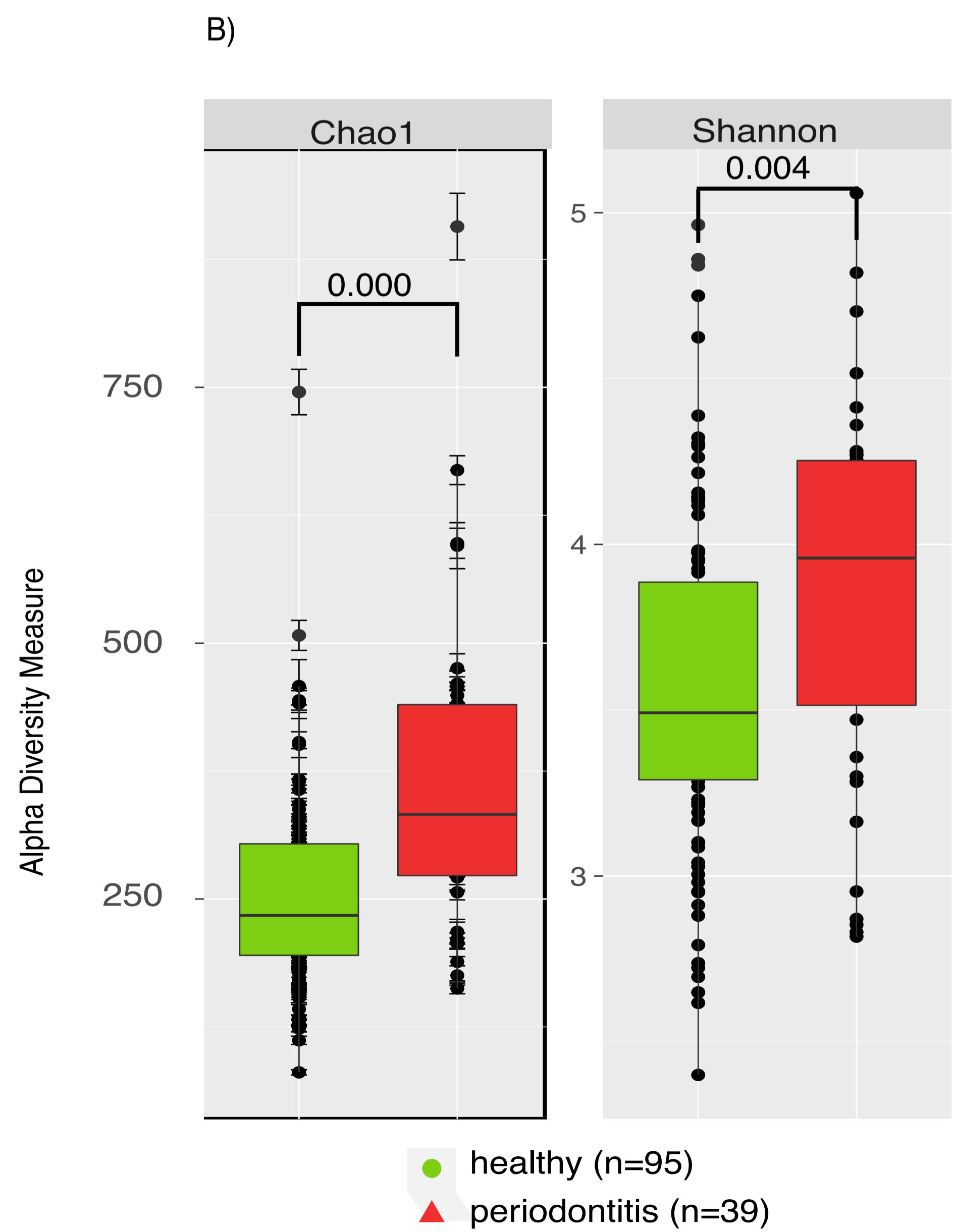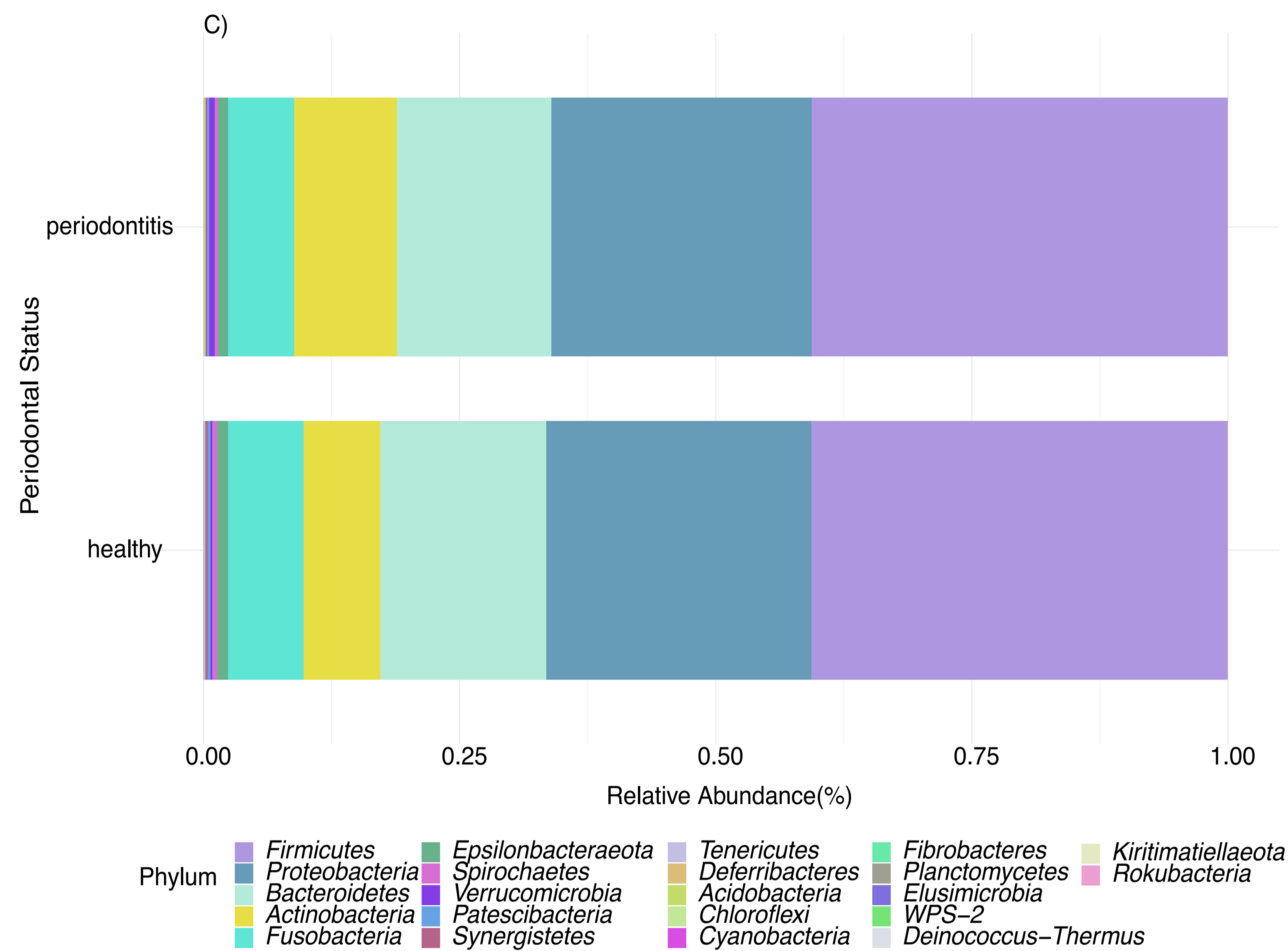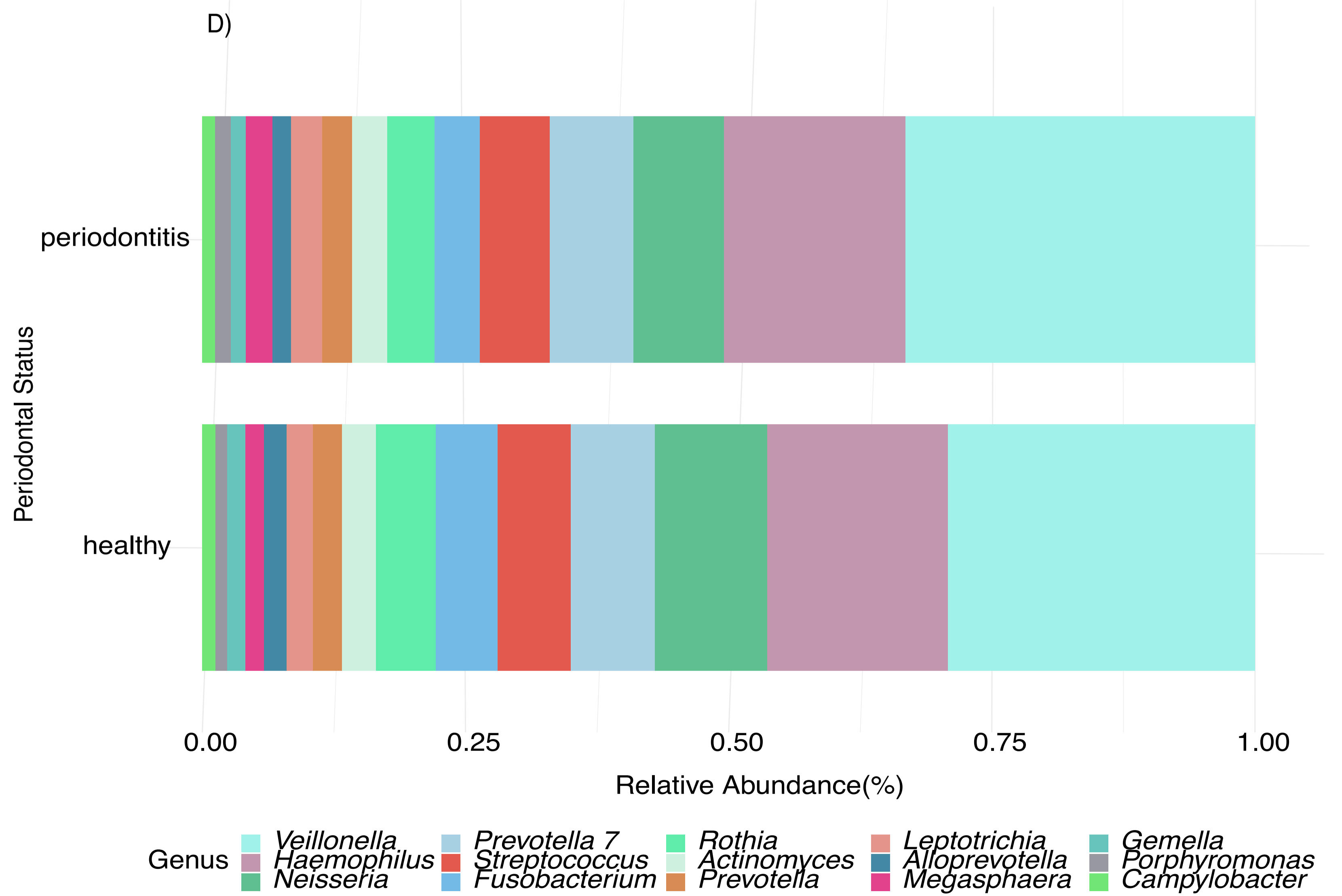

Supplement: Supplementary file 1 [file Image_1.pdf]

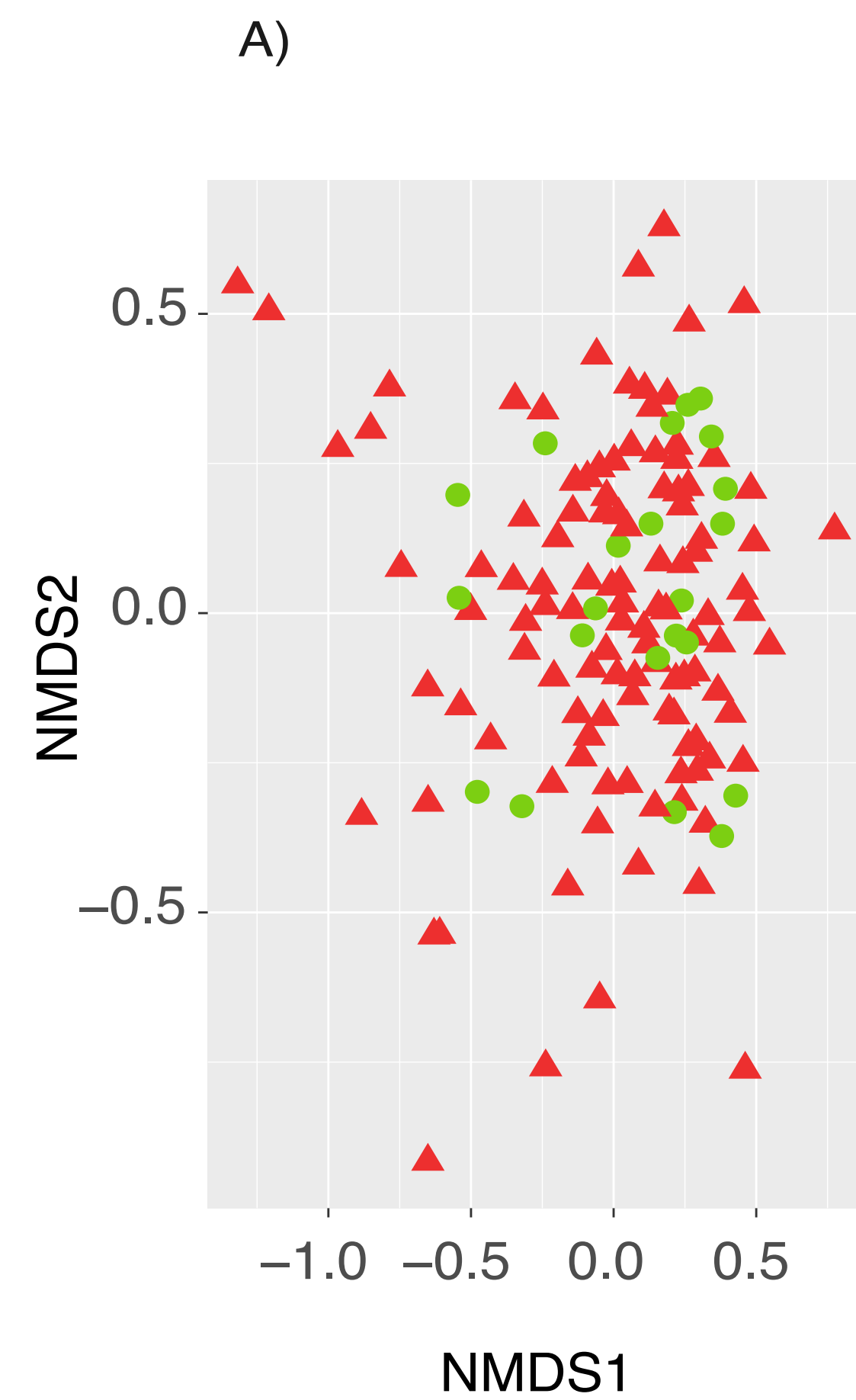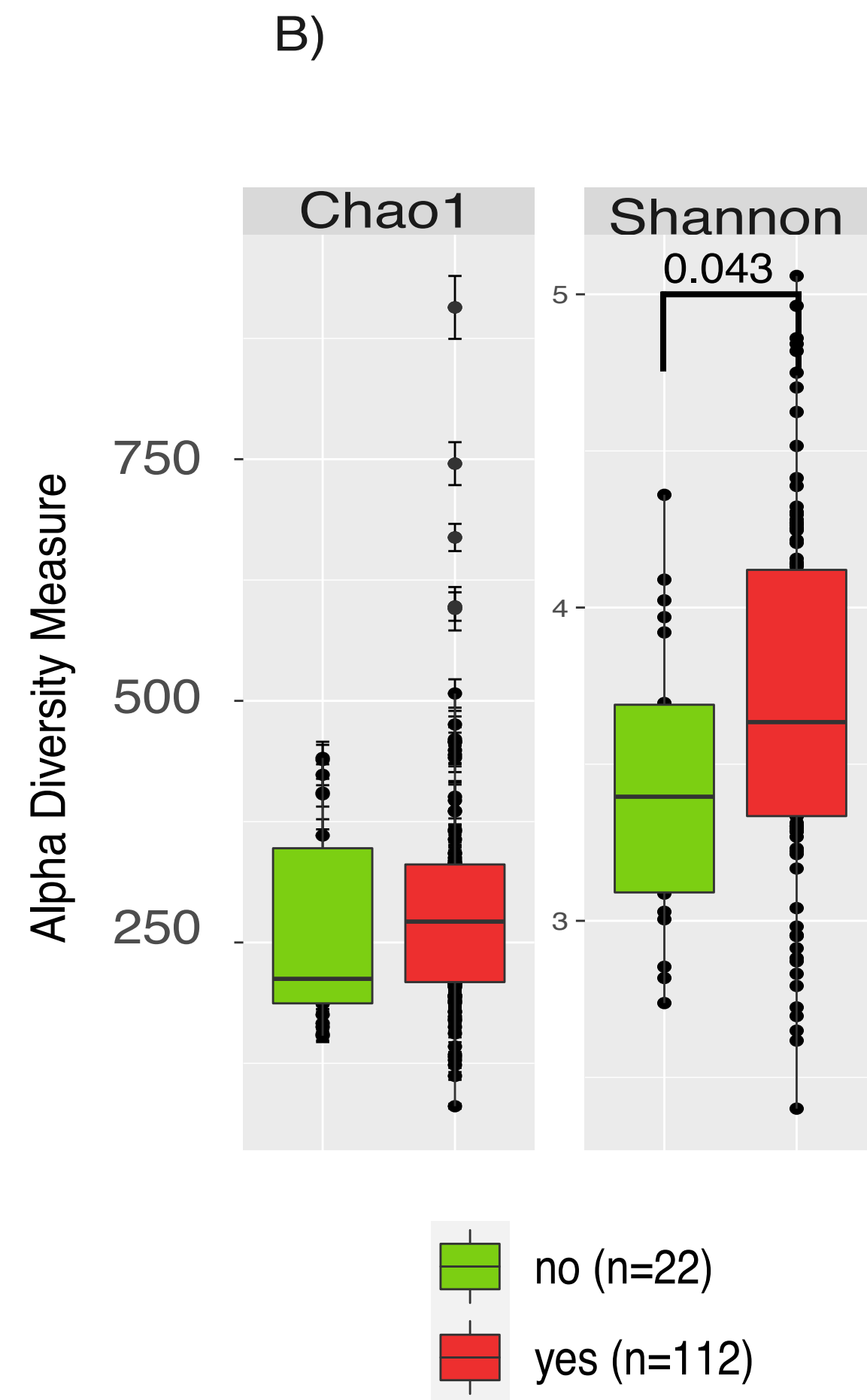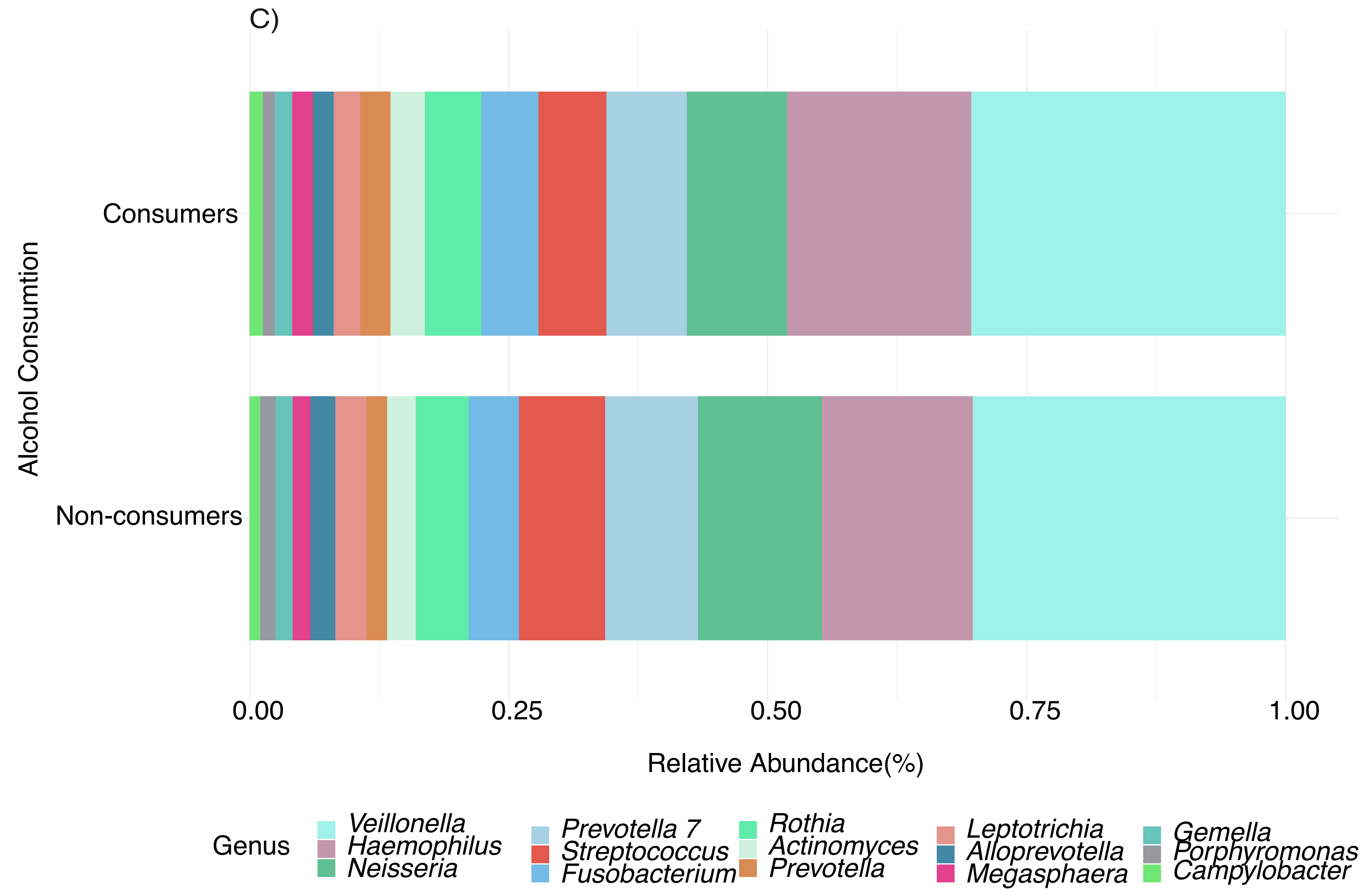

Supplement: Supplementary file 2 [file Image_2.pdf]

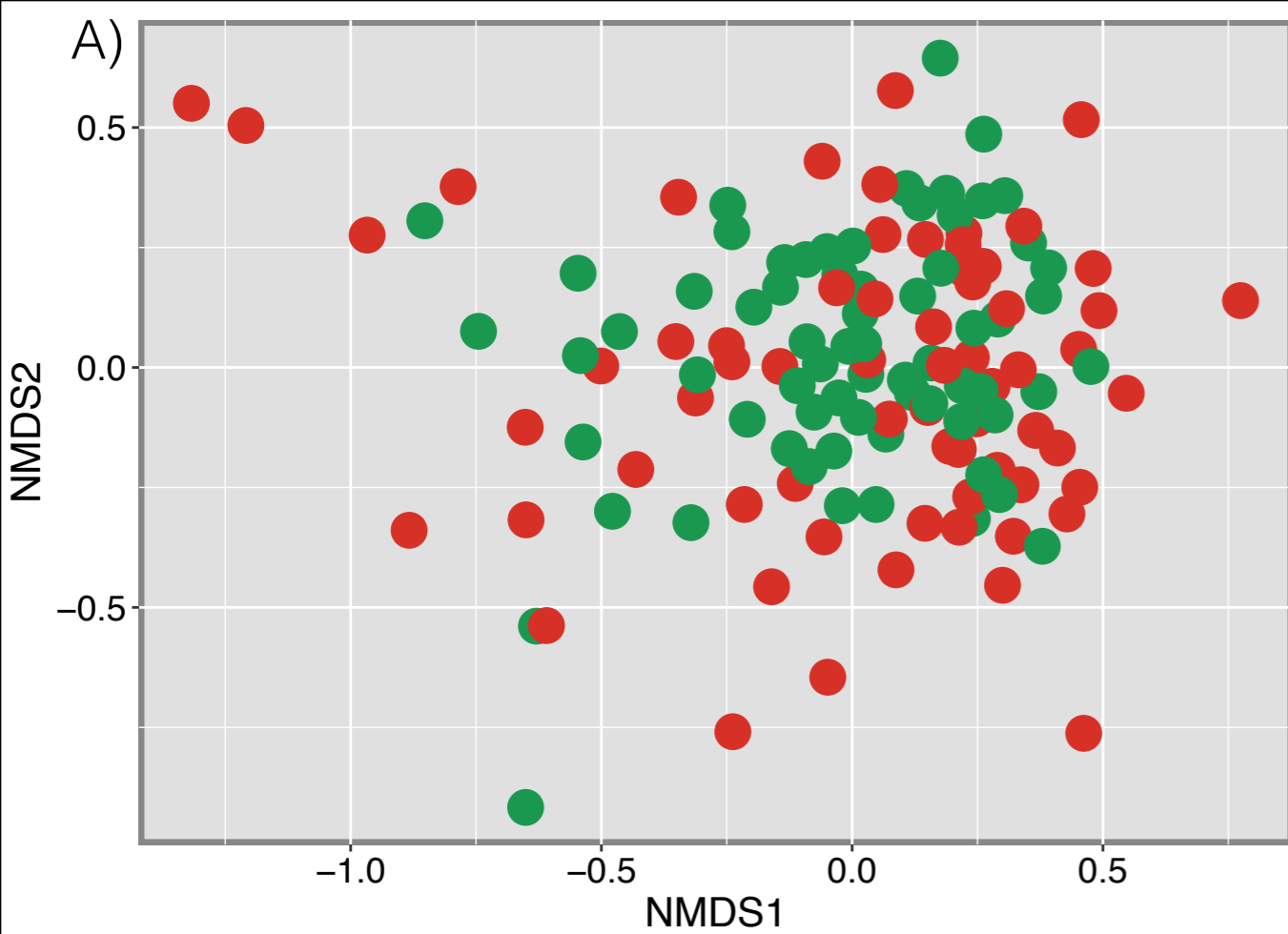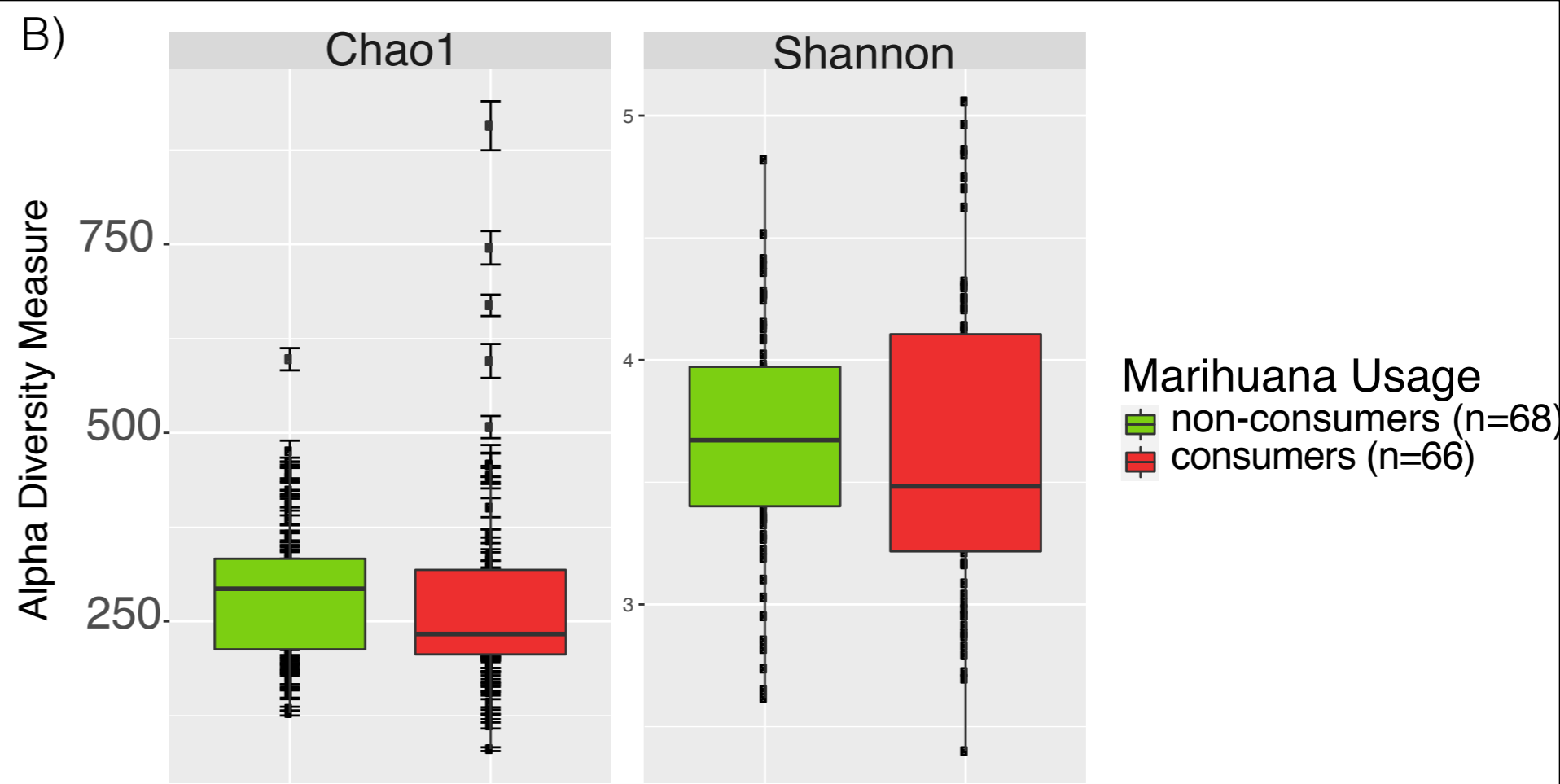

Supplement: Supplementary file 3 [file Image_3.pdf]

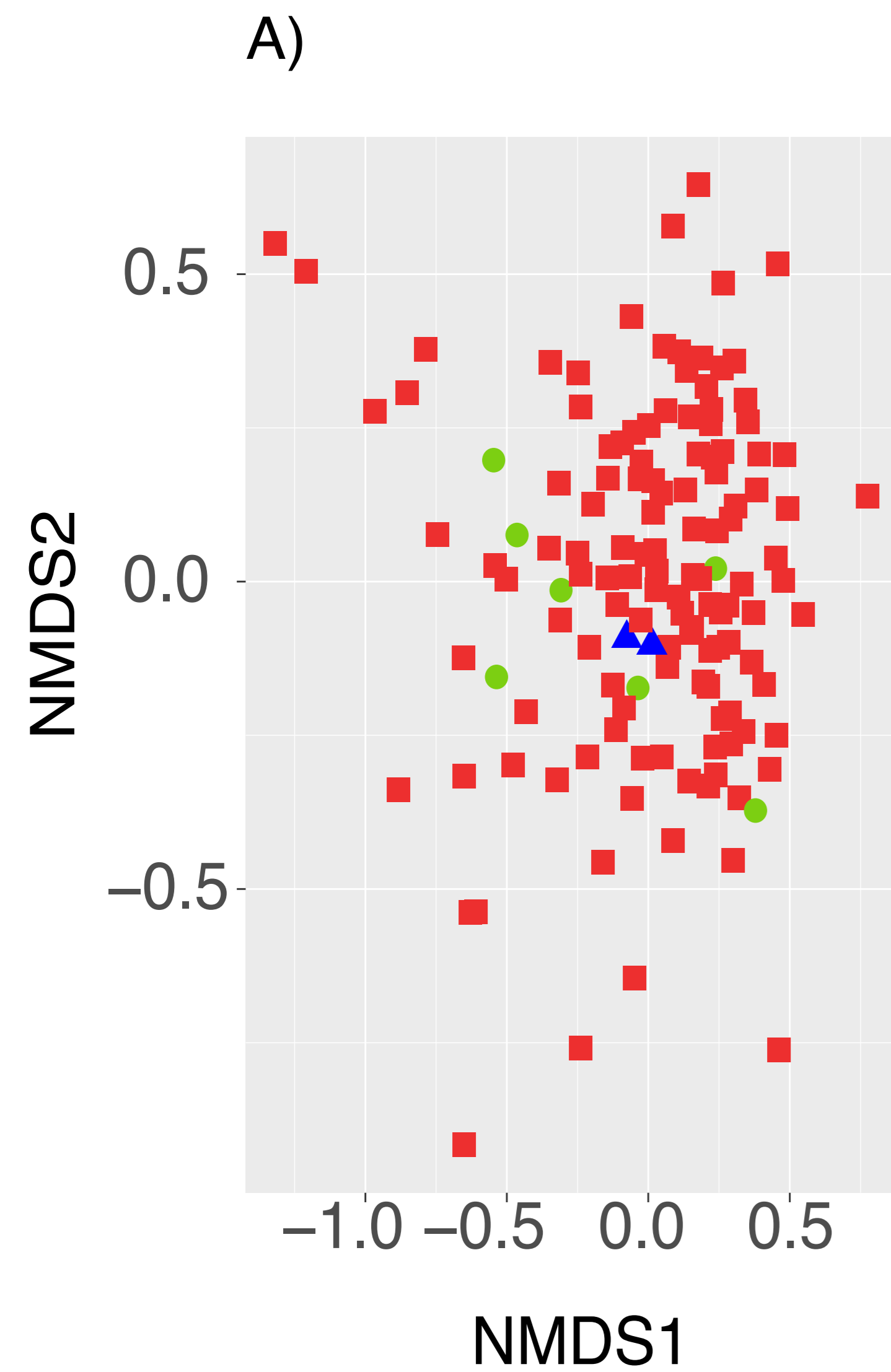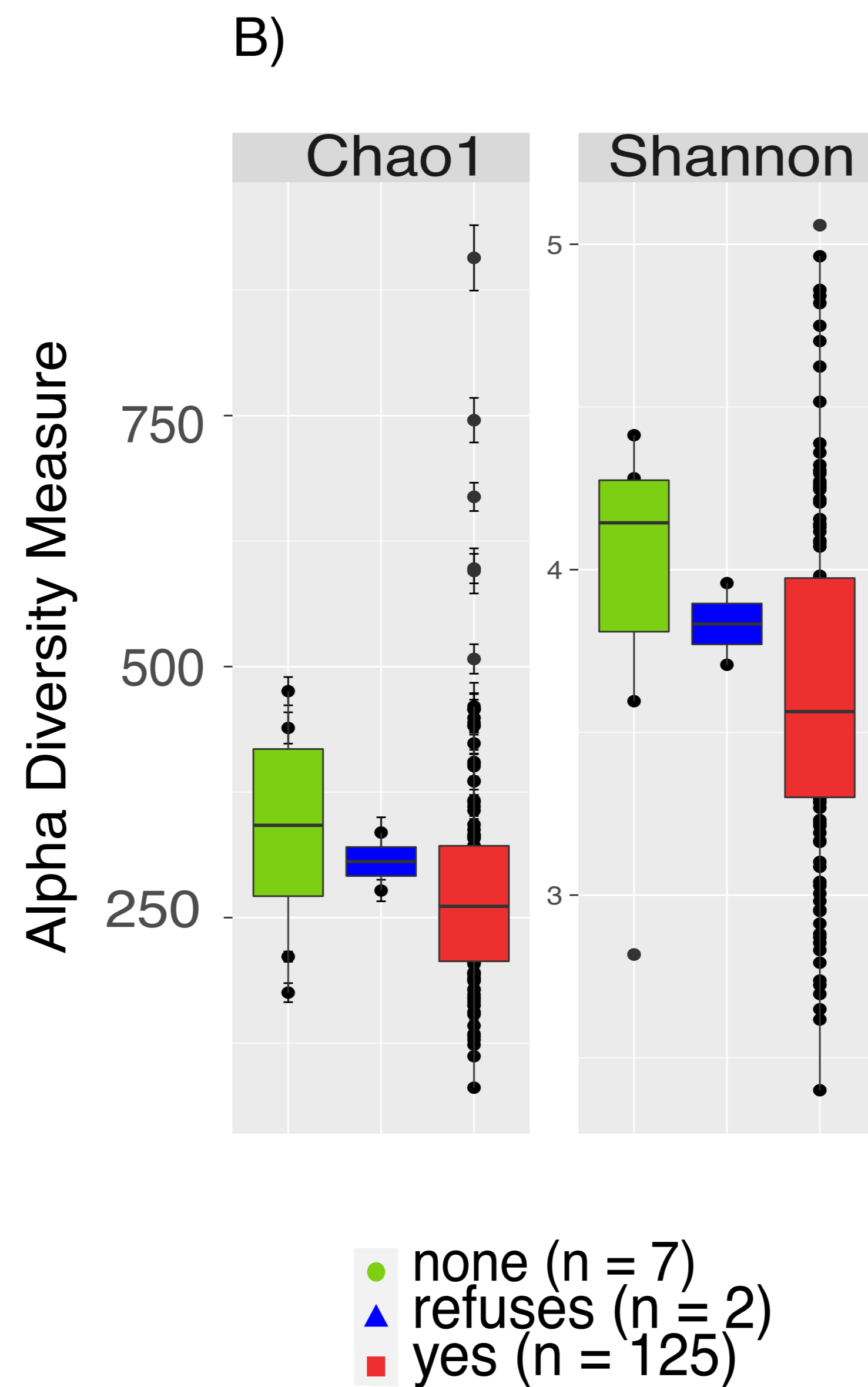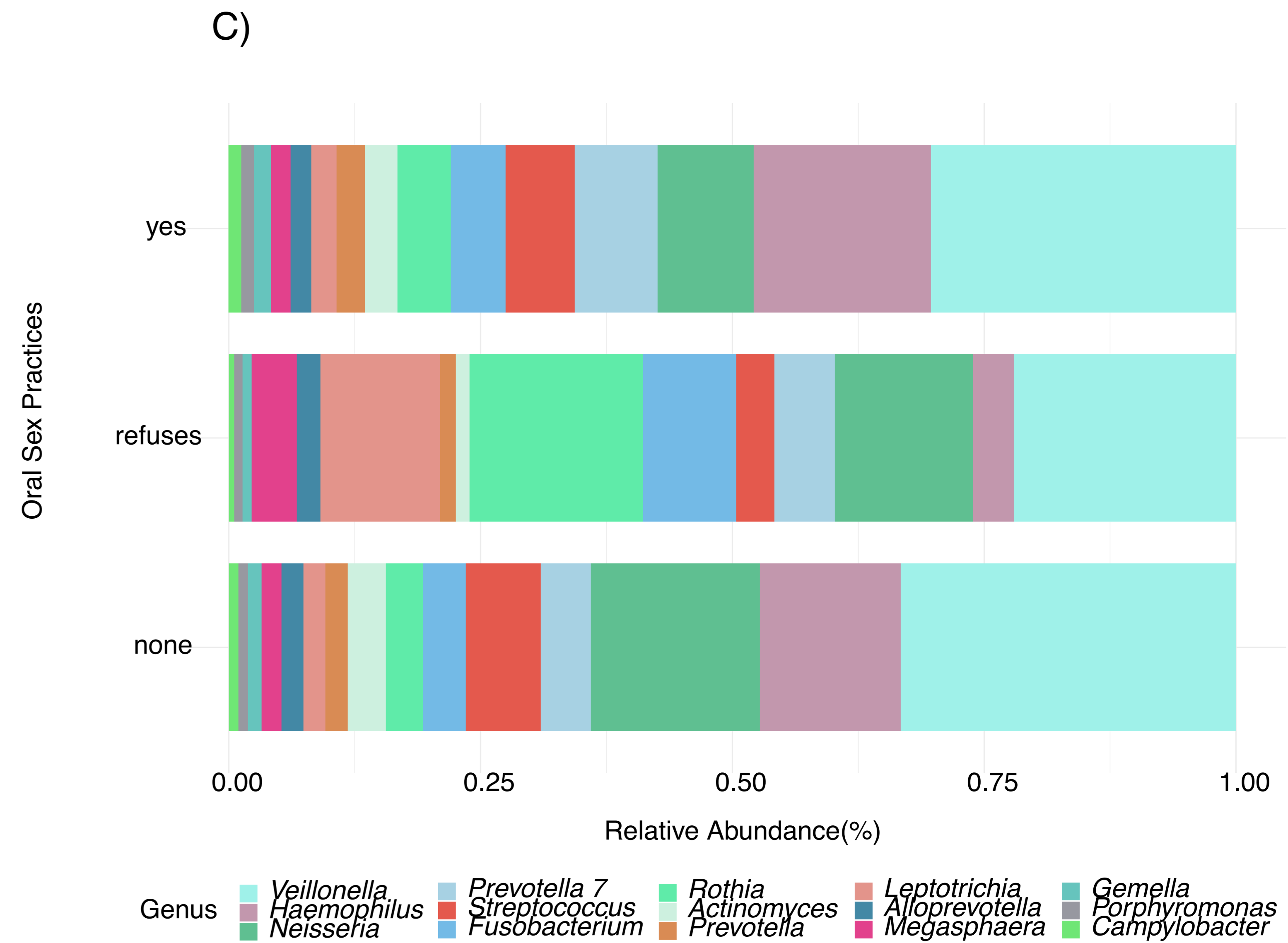

Supplement: Supplementary file 4 [file Image_4.pdf]

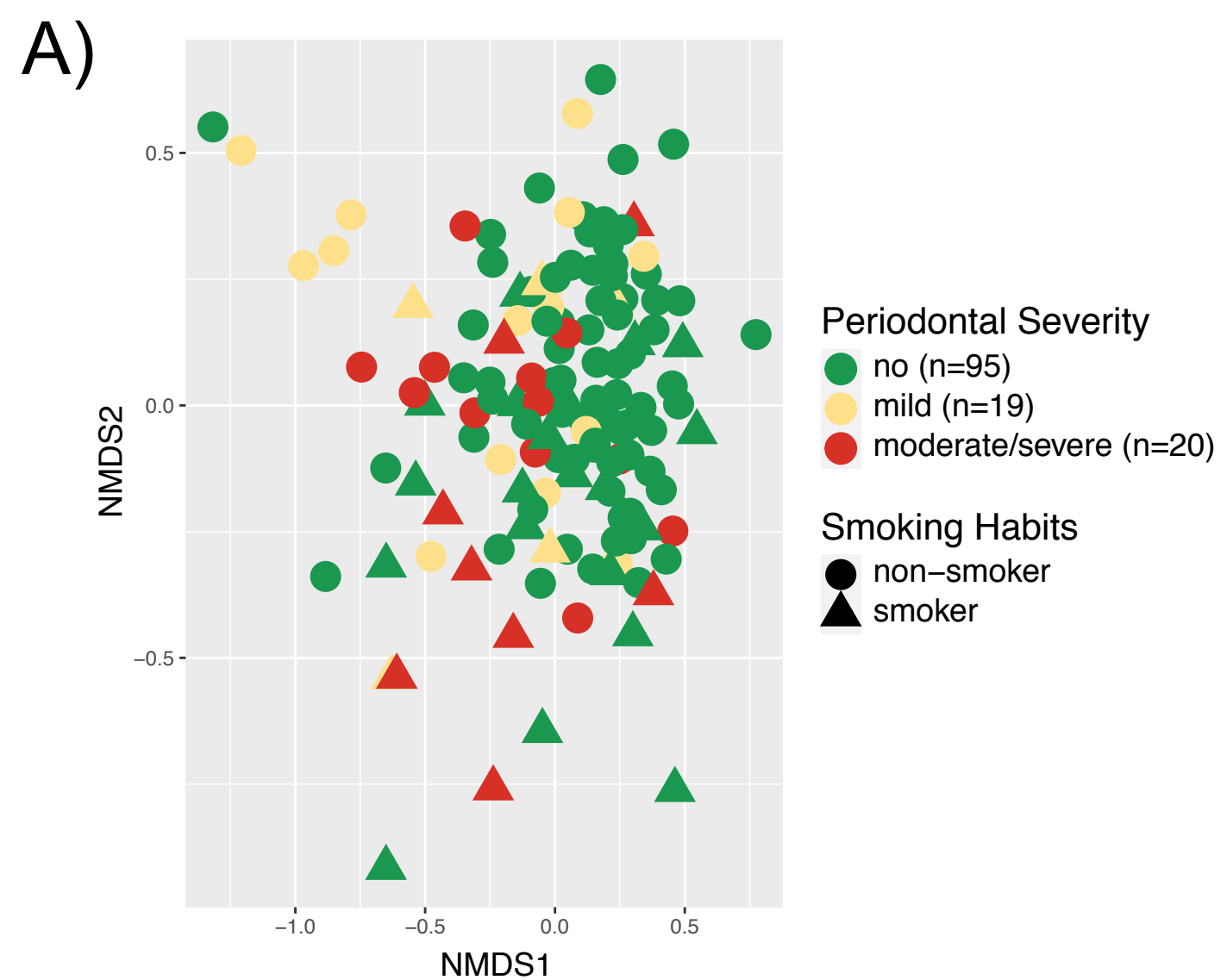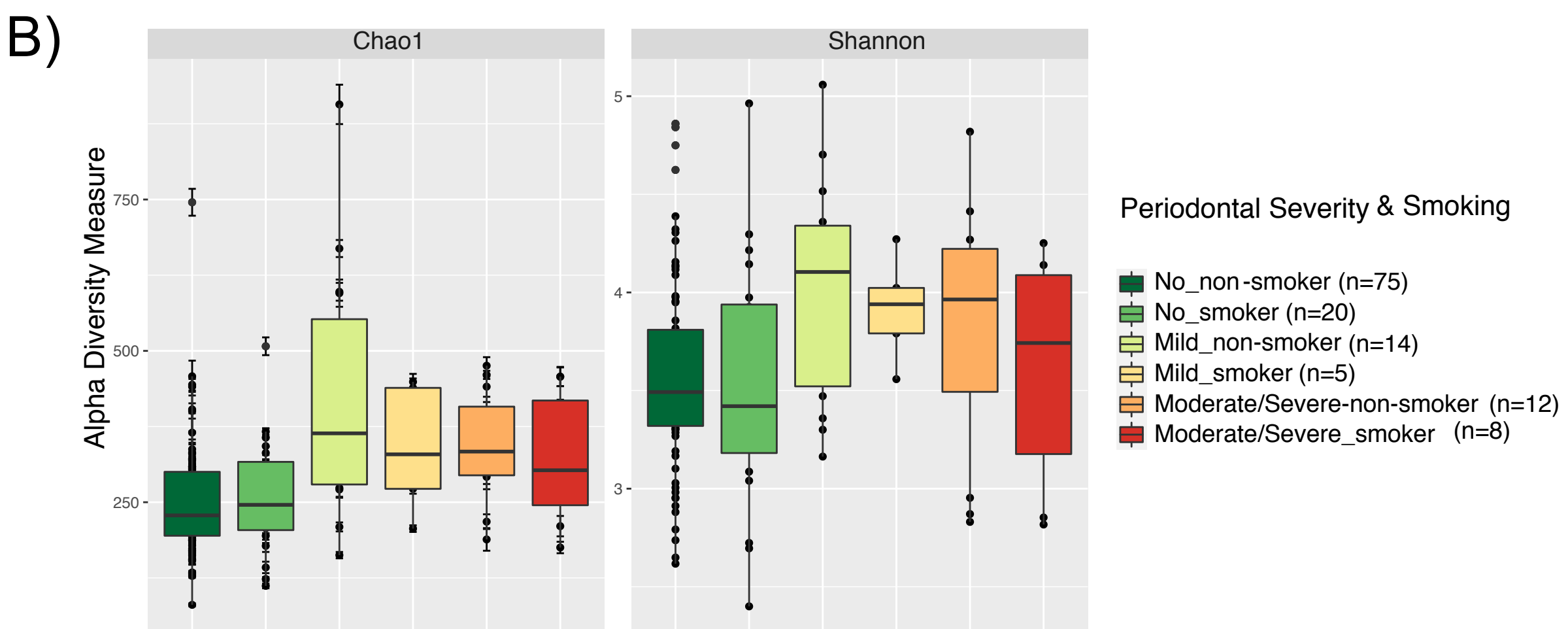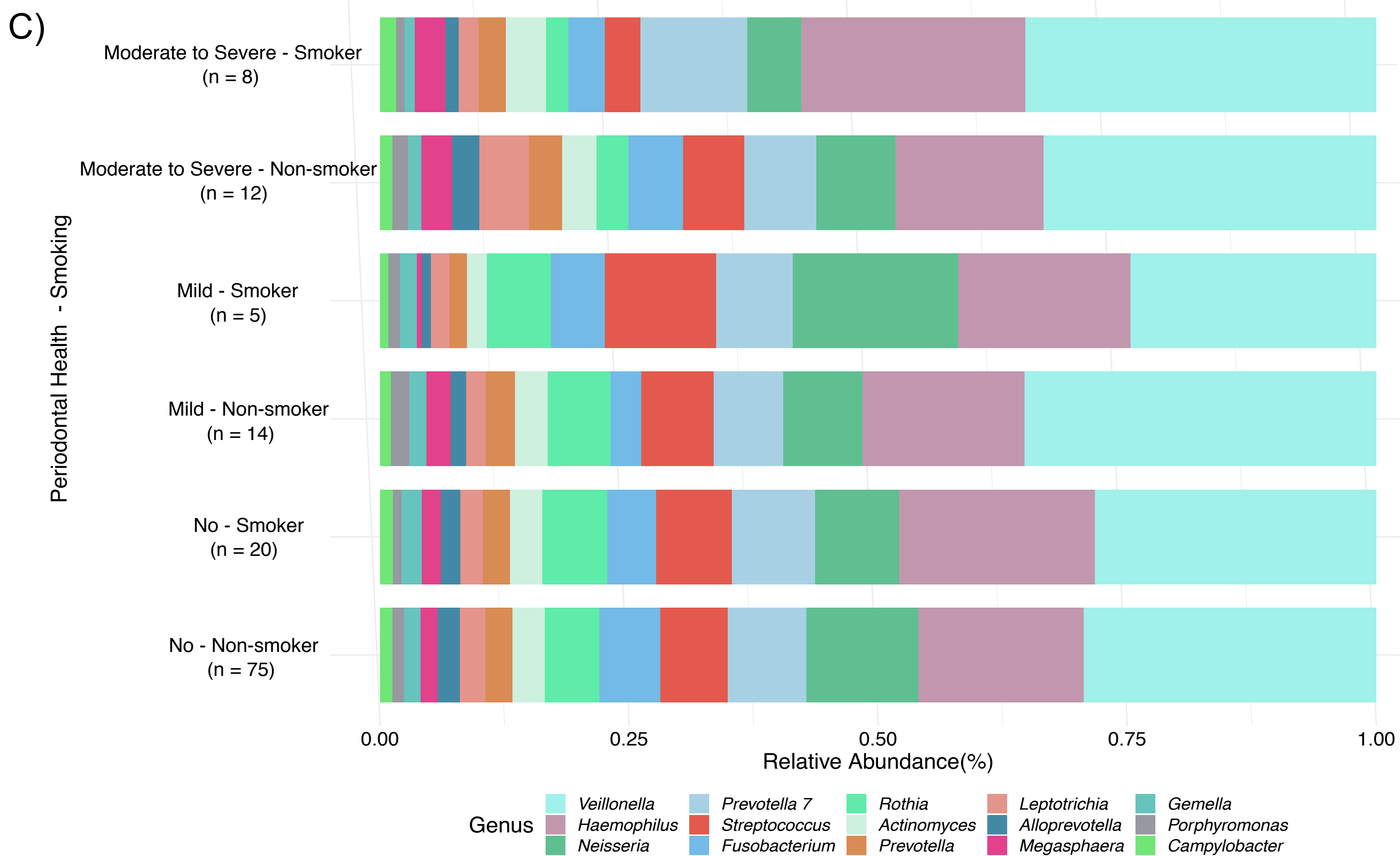

Supplement: Supplementary file 5 [file Image_5.pdf]
